# Evidence for a fisher‐designed solution to manta and devil ray bycatch in tuna fisheries

**DOI:** 10.1111/cobi.70150

**Published:** 2025-10-22

**Authors:** Melissa R. Cronin, Jefferson Murua, Donald A. Croll, Melanie Hutchinson, Nerea Lezama‐Ochoa, Jon Lopez, Hilario Murua, Marta D. Palacios, Victor Restrepo, Joshua D. Stewart, Yonat Swimmer, Kelly M. Zilliacus, Gala Moreno

**Affiliations:** ^1^ Department of Fisheries Oceanography University of Massachusetts Dartmouth New Bedford Massachusetts USA; ^2^ Mobula Conservation La Paz Mexico; ^3^ The Manta Trust Dorchester UK; ^4^ Nicholas School of the Environment Duke University Durham North Carolina USA; ^5^ AZTI Marine Research Basque Research and Technology Alliance (BRTA) Sukarrieta Spain; ^6^ Department of Ecology and Evolutionary Biology University of California, Santa Cruz Santa Cruz California USA; ^7^ Inter‐American Tropical Tuna Commission La Jolla California USA; ^8^ Institute of Marine Sciences Fisheries Collaborative Program University of California, Santa Cruz Santa Cruz California USA; ^9^ International Seafood Sustainability Foundation Pittsburgh Pennsylvania USA; ^10^ Ocean Ecology Lab, Marine Mammal Institute, Department of Fisheries, Wildlife and Conservation Sciences Oregon State University Newport Oregon USA; ^11^ Pacific Islands Fisheries Science Center NOAA Fisheries Honolulu Hawaiʻi USA

**Keywords:** conservation, devil ray, elasmobranch, fisheries, handling and release practices, manta ray, sorting grid, tropical tuna purse seine fishery, knowledge co‐production, conservación, elasmobranquios, mantarraya, pesquerías, prácticas de manejo y liberación, raya diablo, rejilla de clasificación, 软骨鱼, 处理和释放方法, 分拣网格, 保护, 渔业, 蝠鲼, 魔鬼鱼

## Abstract

Bycatch in global tropical tuna purse seine fisheries represents a significant source of mortality for manta and devil rays (mobulids), which are globally threatened. Use of best handling and rapid release practices on purse seine vessels can substantially reduce mortality and improve vulnerability status for mobulids. However, interventions must be effective, operationally feasible, and safe for human operators if they are to be successfully implemented at a large scale. We assessed the feasibility and efficacy of an innovative mobulid bycatch release device (sorting grid) in collaboration with captains and crew of vessels in the tropical tuna purse seine fleet. We evaluated the size of individuals and duration of release when the sorting grid was used and compared these metrics with other release methods. Forty‐one mobulid capture events occurred on 12 vessels. Mobulids released using the sorting grid were significantly larger than those released by other methods; yet, mean handling time remained short (∼3 min), suggesting that the device enables the rapid release of even the largest individuals. Widespread implementation and use of the mobulid sorting grid could help achieve conservation goals for threatened mobulid rays and may have broader bycatch reduction benefits. Adoption of sorting grid requirements by regional fisheries management organizations could standardize best practices and markedly improve the survival of released mobulid rays globally.

## Abstract (es)

Evidencias de una solución diseñada por pescadores para la captura incidental de rayas diablo y mantarrayas en la pesca del atún

**Resumen:**

Las capturas incidentales en las pesquerías mundiales de atún tropical con redes de cerco representan una fuente importante de mortalidad para las mantarrayas y las rayas diablo (mobúlidos), que están amenazadas a nivel mundial. El uso de las mejores prácticas de manipulación y liberación rápida en los buques de cerco puede reducir sustancialmente la mortalidad y mejorar el estado de vulnerabilidad de los mobúlidos. Sin embargo, las intervenciones deben ser eficaces, viables desde el punto de vista operativo y seguras para los operadores humanos si se quieren aplicar con éxito a gran escala. Evaluamos la viabilidad y la eficacia de un innovador dispositivo de liberación de capturas incidentales de mobúlidos (cuadrícula de clasificación) en colaboración con los capitanes y la tripulación de los buques de la flota de cerqueros de atún tropical. Evaluamos el tamaño de los individuos y la duración de la liberación cuando se utilizó la cuadrícula de clasificación y comparamos estas métricas con otros métodos de liberación. Se produjeron 41 capturas de mobúlidos en 12 buques. Los mobúlidos liberados utilizando la rejilla de clasificación eran significativamente más grandes que los liberados por otros métodos, pero el tiempo medio de manipulación siguió siendo corto (aproximadamente 3 minutos), lo que sugiere que el dispositivo permite la rápida liberación incluso de los individuos más grandes. La implementación y el uso generalizados de la rejilla de clasificación de mobúlidos podrían ayudar a alcanzar los objetivos de conservación de las rayas mobúlidas amenazadas y podrían tener beneficios más amplios en la reducción de las capturas incidentales. La adopción de los requisitos de la rejilla de clasificación por parte de las organizaciones regionales de ordenación pesquera podría estandarizar las mejores prácticas y mejorar notablemente la supervivencia de los mobúlidos liberados en todo el mundo.

## 渔民设计的减少金枪鱼捕捞中蝠鲼兼捕的解决方案的证据

**【摘要】** 导致蝠鲼死亡的一个重要原因是全球热带金枪鱼围网渔业中发生的兼捕, 而该类群已在全球范围内濒危。在围网渔船上采用最佳处理和快速释放方法可大大降低蝠鲼的死亡率, 并帮助改善其濒危现状。然而, 要想成功地大规模推广干预措施, 这些措施必须有效、易于操作, 且能保障操作人员的安全。我们与热带金枪鱼围网船队的船长和船员合作, 评估了一个创新的蝠鲼兼捕释放装置(分拣网格)的可行性和有效性。我们评估了使用分拣网格时的个体大小和释放所需时间, 并将这些指标与其他释放方法进行了比较。研究共记录了12艘船上发生了41起蝠鲼兼捕事件。使用分拣网格释放的蝠鲼个体明显大于其他方法释放的个体, 而平均处理时间仍较短(约3分钟), 这表明该装置能实现最大个体的快速释放。蝠鲼分拣网格的推广和使用有助于实现受威胁蝠鲼的保护目标, 并有利于更广泛的减少兼捕。由区域渔业管理组织来要求采用分拣网格, 可以推动最佳实践的标准化, 并显著提高全球被释放蝠鲼的存活率。**【翻译:胡怡思;审校:聂永刚】**

## INTRODUCTION

Manta and devil rays (mobulids) are an iconic group of slow‐growing, filter‐feeding rays (Couturier et al., 2012). Found globally in tropical and subtropical waters, the ten described mobulid species range widely in size, from Munk's devil ray (*Mobula munkiana*) (up to 1.1 m disc width) to the oceanic manta ray (*Mobula birostris*) (up to 7.1 m disc width) (Couturier et al., 2012; Stewart et al., 2018). Mobulids exhibit some of the lowest reproductive rates among sharks and rays due to their extremely low annual reproductive output (∼one pup every 1–3 years), long gestation period (∼12 months), and delayed maturation (∼7–10 years), making them especially vulnerable to anthropogenic pressures such as overexploitation (Couturier et al., 2012; Deakos, 2012; Dulvy et al., 2021; Fernando & Stewart, 2021; Marshall & Bennett, 2010; Pardo et al., 2016; Stevens, 2016). Although the lifespan of mobulids remains largely uncertain, it is estimated that *M. birostris* can live for at least 40 years (Stevens et al., 2018). Data on mobulids are extremely scarce because they are generally nontarget species in large‐scale fisheries, are distributed in pelagic areas, and interact with fisheries that are difficult to access and monitor (Ward‐Paige et al., 2013). As a result, estimates of many important life‐history parameters are missing for most mobulid species (Pardo et al., 2016; Stewart et al., 2018). Given their sensitive life‐history characteristics and limited available data, mobulids are classified among the most threatened elasmobranch species (Dulvy et al., 2008, 2021). Although species‐specific population data are limited, significant declines in local and regional populations have been documented in all oceans, with some areas reporting declines of over 90% (Moazzam, 2018; Rohner et al., 2017; Venables et al., 2024; Ward‐Paige et al., 2013). As a result, all recognized mobulid species are listed as endangered or vulnerable on the International Union for Conservation of Nature Red List of Endangered Species and are listed in Appendix II of the Convention for International Trade in Endangered Species, which regulates their international trade (CITES, 2025; IUCN, 2025).

Bycatch, or incidental capture in fisheries, is one of the most widespread threats to marine megafauna globally, contributing significantly to declines in populations of sharks, rays, marine mammals, sea turtles, and fishes (Lewison et al., 2014). Its impacts are especially pronounced for large, slow‐reproducing species, which are vulnerable to even low levels of incidental mortality (Komoroske & Lewison, 2015). Mobulid populations are globally affected by bycatch, and are caught in at least 30 fisheries of different gear types in 23 countries (Croll et al., 2016). Artisanal fisheries contribute substantially to mobulid target and bycatch mortality. However, these fisheries are data‐poor, highly decentralized, and it is challenging to address conservation measures associated with them (Alfaro‐Cordova et al., 2017; Fernando & Stewart, 2021). The tropical tuna purse seine fishery is an important source of mobulid bycatch; thousands of individuals are captured annually. However, reliable, recent global estimates of mobulid bycatch in tuna purse seine fisheries do not exist (Croll et al., 2016; Lezama‐Ochoa et al., 2019).

The largest tropical tuna purse seine fishery is in the Pacific Ocean, where more than half of the global tuna purse seine catch occurs (Justel‐Rubio, 2024). In contrast to other fishing gears and fisheries, vessels operating in the western and central Pacific Ocean and the largest vessels (>363 metric tons carrying capacity) in the eastern Pacific currently have 100% observer coverage, allowing for more accurate assessments of bycatch (Hall & Roman, 2013). Purse seine fisheries are governed by tuna regional fisheries management organizations (tRFMOs), which means conservation interventions have the potential for wide impact (Dulvy & Simpfendorfer, 2022). Though the rate of species‐specific mobulid interactions with tropical tuna purse seine fishing is not well understood, simulations of variable management scenarios suggest that reducing postrelease mortality could significantly improve the likelihood of survival and therefore the status of mobulid populations (Griffiths & Lezama‐Ochoa, 2021; Stewart et al., 2024).

**FIGURE 1:**
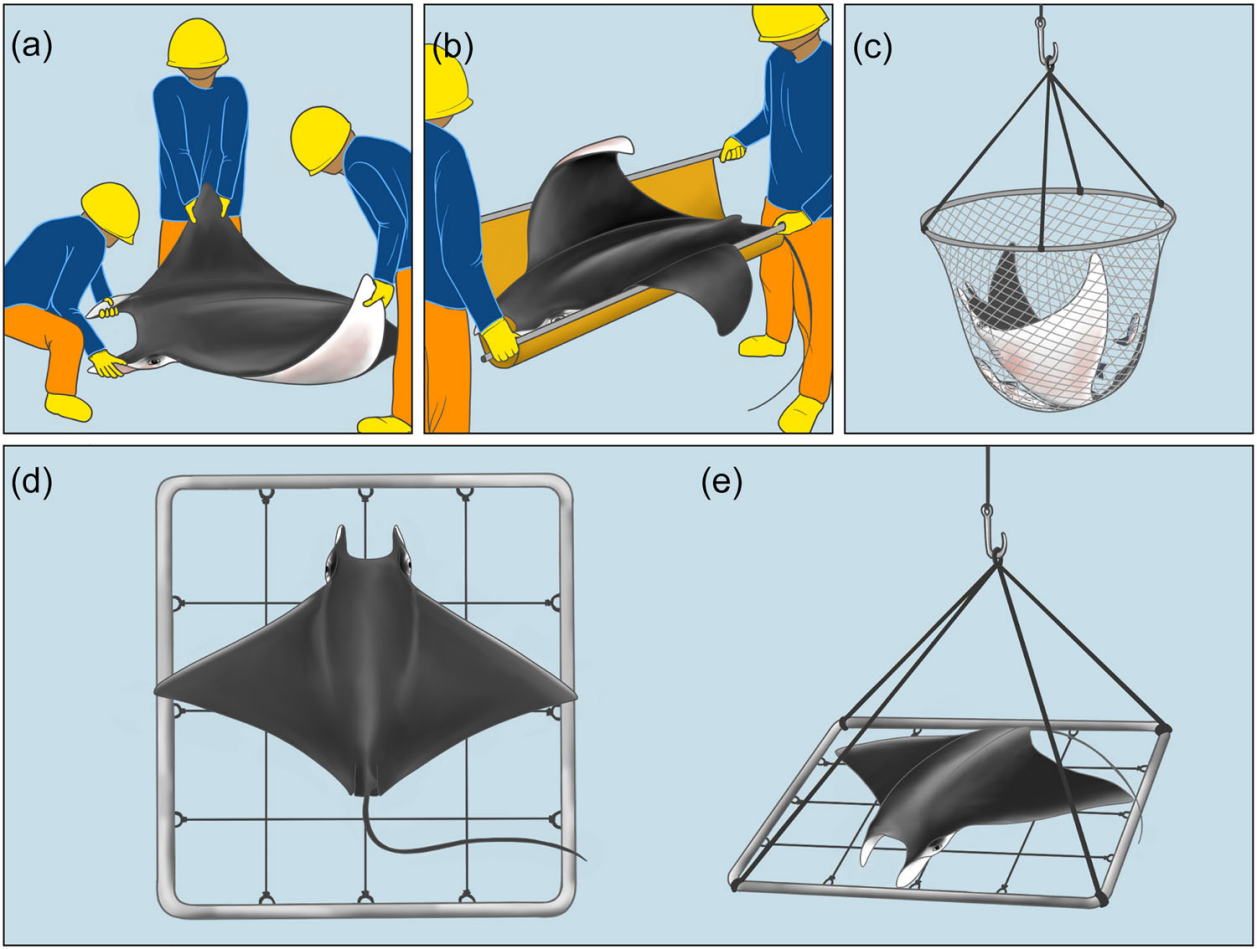
Variations in handling and release methods for large mobulids captured in tuna purse seine vessels: (a) manual release, (b) stretchers, (c) brailer scoop nets, and (d, e) the mobulid sorting grid. Illustrations by Julie Johnson, Life Sciences Studios.

Currently, handling and release practices for mobulids vary widely among purse seine vessels (Cronin et al., 2022) (Figure 1). Generally, mobulids are brought onto the deck along with tuna in a large, hydraulicly powered scoop net called a brailer and are then manually released by crew, though this can result in damage to the delicate cephalic lobes or eyes (Figure 1a) (Murua et al., 2021). Further, these methods cannot be used for very large individuals, which can be left on the deck during the fishing operation, risking asphyxiation, crushing of internal organs, and mortality (Cronin et al., 2022). In the past, fishers would insert hooks in the gill slits, pectoral fins (wings), or both of large mobulids to lift and release them with the vessel's crane (which likely fatally injured most animals), a practice that is currently banned by all tRFMOs (Hall & Roman, 2013; Hutchinson et al., 2023, 2017). Recently, crews have begun using simple tools like stretchers, cargo nets, or the brailer to release large individuals overboard (Figure 1c) (Poisson et al., 2014). However, these tools can result in severe bending of the wings, with undocumented impacts on survival (Hutchinson et al., 2020; Murua, Zudaire, et al., 2023). These large individuals also still need to be manually extracted from the brailer, which can be difficult and time‐consuming, resulting in the animal being left on deck until there is an opportunity to allocate resources and crew to release it (Murua, Ferarios, et al., 2023; Murua, Moreno, et al., 2023).

Recent research synthesizing satellite tag data across multiple oceans has demonstrated that mobulid survival rates following bycatch in tuna purse seine fisheries are strongly influenced by time spent on deck, with release under three minutes being a critical target for higher likelihood of survival, underscoring the urgent need for handling and release methods that facilitate their rapid release to sea (Stewart et al., 2024). Addressing this issue requires the development and evaluation of new release techniques for quickly returning mobulids to sea without injury, particularly for larger individuals, to enhance postrelease survival rates.

Addressing bycatch in tuna purse seine fisheries is critical for mobulid conservation globally, given these species’ ecological importance and vulnerability. We sought to document steps in the deployment and evaluation of bycatch release devices (BRDs) in the form of mobulid sorting grids on vessels in the US tropical tuna purse seine fleet in the Pacific Ocean, including an estimation of mobulid capture and release times (i.e., time out of the water). Additionally, we describe progress, challenges, and lessons learned from the collaborative design, deployment, and testing of BRDs involving fisher participation.

## METHODS

### Participatory approach to the evaluation of BRDs

**FIGURE 2:**
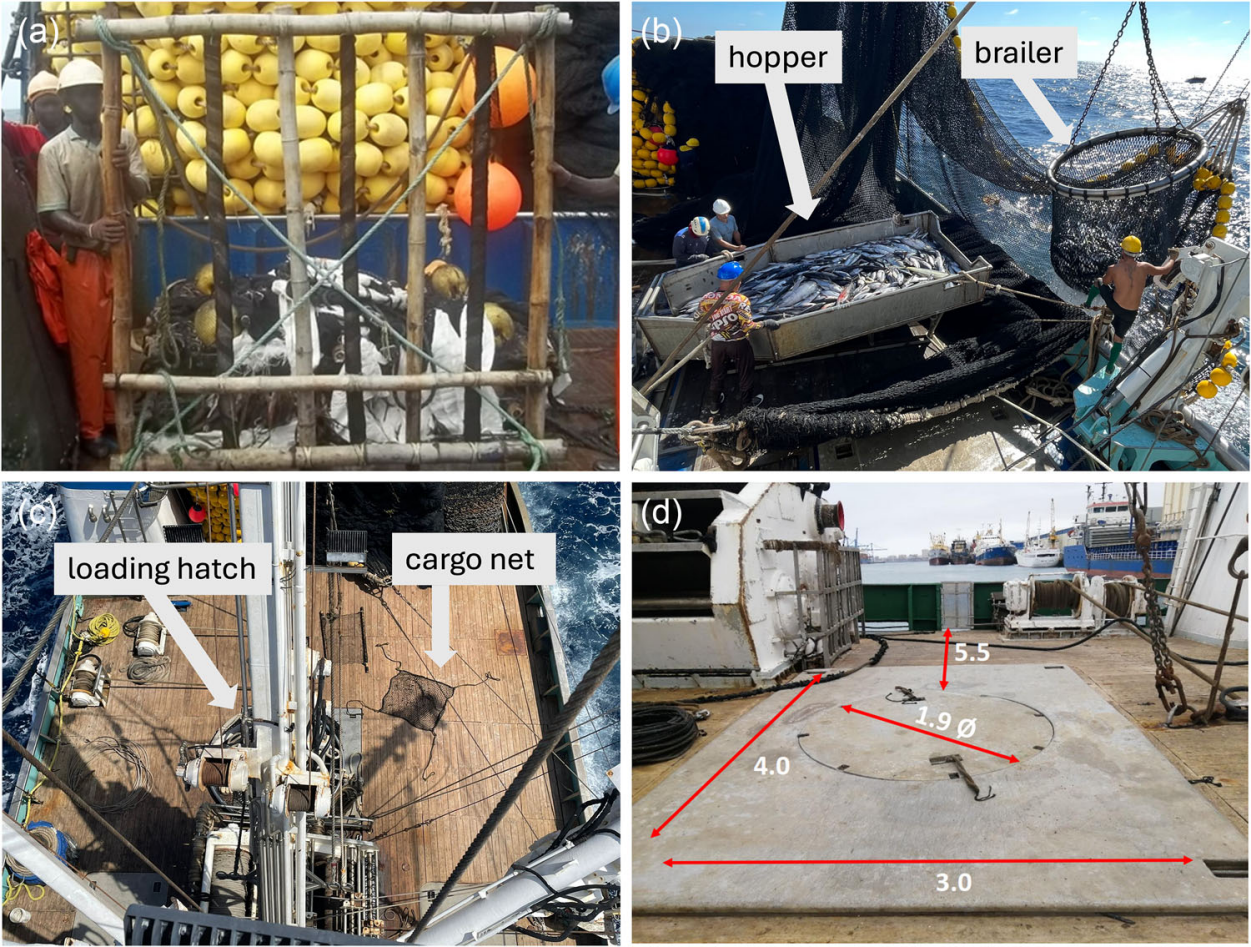
(a) Original prototype of the mobulid sorting grid, a bamboo grid constructed by fishers on a Spanish vessel in the Atlantic Ocean (photo by Atunsa), (b) hopper used to sort bycatch by some vessels and brailer used to scoop catch and bycatch onto the vessel deck (photo by M.R.C.), (c) working deck of the *F/V Captain Vincent Gann*, a U.S. tuna purse seine vessel, and (d) measurements of the loading hatch provided by fishers to enable customization of the sorting grid (photo provided by the American Tunaboat Association).

Involving fishers in the development and adoption of BRDs from the outset is essential to encourage adoption and make them successful as a conservation tool. Fishers’ practical knowledge and hands‐on experience with gear and fishing operations provide valuable insights that can shape the feasibility, design, testing, and refinement of these tools (Murua, Moreno, et al., 2023). A notable example is the mobulid sorting grid, which was originally conceived by a fisher in the Atlantic Ocean working for a Spanish fishing company (Atunsa). During a workshop on best fishing practices, this fisher described how they used bamboo, a material commonly found onboard for constructing fish aggregating devices (FADs), to construct a grid to release large marine species like mobulids and sharks (Figures 1d,e & 2a) (Murua, Zudaire, et al., 2023). Scientists recognized the potential of this fisher‐driven innovation for purse seine fisheries and started trialing them in diverse tuna purse seine fleets (Murua et al., 2024). The original bamboo design was improved through the use of more durable materials, such as stainless‐steel tubes, and a release process with easily cut thick ropes.

We assessed the feasibility and efficacy of the mobulid sorting grid, in collaboration with the captains and crew of vessels from the U.S. tropical tuna purse seine fleet. The U.S. tropical tuna purse seine fleet consists of 12 vessels targeting primarily skipjack (*Katsuwonus pelamis*), yellowfin tuna (*Thunnus albacares*), and bigeye tuna (*Thunnus obesus*) across the tropical Pacific Ocean, on the high seas, and within the exclusive economic zones of some Pacific Island countries (NOAA, 2024).

Mobulid bycatch estimates do not exist for the entire Pacific and are not available at the fleet or vessel level. However, tRFMO data indicate that in the eastern Pacific Ocean (∼227 vessels), estimated average annual mobulid bycatch across all fleets and set types by large purse seine vessels (carrying capacity >363 metric tons) was approximately 1897 individuals per year from 1993 to 2023 (range 485–6536) (IATTC, 2024a). In 2023, the total number of individuals reported was 755. In the western and central Pacific Ocean (∼305 vessels), estimates suggest an average mobulid bycatch of 3122 individuals per year from 2003 to 2020 (Peatman, 2021; ISSF, 2024a). These estimates represent bycatch only for large vessels—those with 100% observer coverage—and are not specific to the U.S. fleet, which was the subject of our study.

To involve the fishers in the design and evaluation of the mobulid sorting grid, we conducted virtual meetings with ship owners, fleet managers, and crew and in‐person meetings onboard purse seine vessels during port visits to American Samoa and Ecuador. We shared documents explaining mobulid sorting grid characteristics and videos showing releases by crew members using this BRD in purse seiners with the fleet during these meetings to illustrate correct usage.

### Sorting grid construction and functioning

To define and test safe‐handling and release practices, mobulid sorting grids designed by AZTI‐Tecnalia (Murua et al., 2024) were constructed by crew on each of the 12 participating purse seine vessels in the U.S. flagged tuna purse seine fishery. Generally, the mobulid design consists of a rigid frame, typically made of stainless steel, with cords or ropes arranged in a grid (Appendix S1). The grid is placed over the hopper (a steel tray with lateral walls where the contents of the brailer can be spread out for inspection prior to release into the loading hatch) (Figure 2b) or directly over the vessel's loading hatch, through which tunas are transferred to the freezer hold (Figure 2d). The large size of the grid squares allows tuna to pass through into the loading hatch while preventing the larger mobulid from falling through the hatch. The sorting grid can be attached to an existing hydraulic crane on the vessel, enabling animals landing in the grid to be lifted and released overboard without manual labor. Each tuna purse seine vessel features a unique deck design and configuration, including differences in deck size, the presence or absence of a hopper, and other variations. To ensure the effective design of sorting grids for each vessel, fishers were asked to complete a questionnaire (Appendix S2) detailing the specific characteristics of the deck of their vessel and the maneuvers used in deck operations during landing. We also asked fishers to provide photographs of their vessel decks (Figure 2c) and accurate measurements of the loading hatch (Figure 2d). These data enabled the customization of mobulid sorting grid designs to fit the unique specifications of each vessel.

Once customized mobulid sorting grid designs for each vessel were developed, fishing crew constructed the sorting grids themselves. This hands‐on approach allowed them to work directly with the device and to use materials typically available onboard (such as stainless‐steel pipes and ropes) and promoted familiarity with the tool. Additionally, this method allowed fishers to make adjustments and improvements to the device based on their own practical experience. An additional benefit from this participatory process was that in the event of future damage to the sorting grid, their firsthand experience would allow them to easily repair it.

### Data collection

Mobulid captures were documented on 12 vessels (all large vessels, with capacity greater than 363 tons). Three 2‐month trips with one scientist onboard each were taken from December 2022 to February 2023, May to June 2023, and March to April 2024, respectively. To extend sampling coverage beyond these trips, in‐person trainings were conducted to instruct fishers on data collection protocols. Additional workshops were conducted at port and during cruises to improve species identification and knowledge of proper use of the grid, as well as handling and release methods. Fishing crew (nonobservers) were trained to record capture and release data on a standard form to collect the same data points as scientist‐collected data. Crew were instructed to use the sorting grid whenever possible, though data were still collected when the grid was not used. Crew collected data from March 2022 to October 2024. Methods used in this study followed the Institutional Animal Care and Use Committee of the University of California, Santa Cruz (UCSC IACUC) Crold2102.

All vessels fished primarily on fish associated with FADs and, to a lesser extent, schools of fish that were swimming freely (unassociated sets). Data collected for each capture included time, location, species, disc width, number sequence of brailer (which can be used as an indicator of the amount of time an animal remains enclosed in the sacked net under the water during the fishing operation) (Figure 2b), duration of capture (from the time the mobulid emerges in the brailer to the time of release), release method (e.g., sorting grid, manual, stretchers), and composition and amount of tuna catch.

### Data analyses

To provide a simple assessment of the effectiveness of the mobulid sorting grid in quickly releasing larger mobulids, we compared the disc width (width wing tip to wing tip, a proxy for mobulid body size) for individuals released using the sorting grid versus other release methods. For this analysis, we filtered the data to exclude captures released directly from the fishing net prior to reaching the vessel deck; however, these cases were described separately in the results. We also excluded individuals with incomplete data for release duration, release method, or size data; sample sizes are reported for each analysis. This was necessary because, although crew‐led data collection expanded the temporal and spatial coverage of the dataset, crew‐collected records were occasionally incomplete due to challenging field conditions and the limited scientific experience of crew members. Remaining individuals were divided into 2 groups: those released using a sorting grid and those released without sorting grid. Shapiro–Wilk tests assessed normality within each group. Depending on normality, either Wilcoxon rank‐sum tests or Welch's 2‐sample *t*‐tests were used to compare the mean disc widths and capture durations between groups using R 4.4.1.

Given the expectation of small sample sizes and the importance of assessing the reliability of detected effects, a post hoc power analysis was conducted to evaluate whether the data showed significant differences between groups. Using Cohen's *d* as the measure of effect size and a significance level of 0.05, this analysis provided an estimate of statistical power based on the observed effect sizes.

## RESULTS

### Sorting grid implementation

**FIGURE 3:**
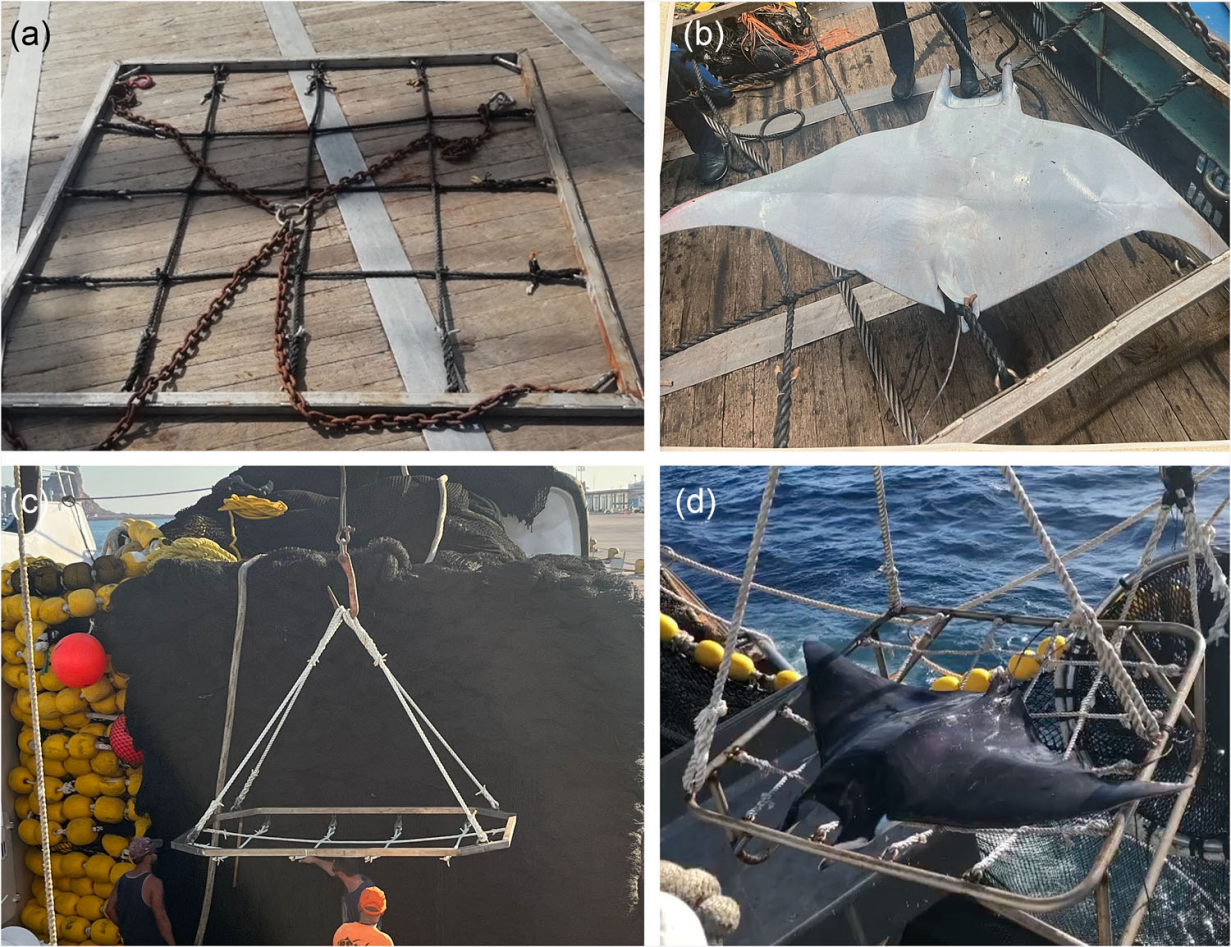
Sorting grids designed and deployed on U.S. tuna purse seine vessels: (a) bungee grids and chain suspension system (photo by J.M.), (b) flexible rope grid (photo provided by American Tunaboat Association), (c) tapered corner grid (photo by M.R.C.), and (d) raised grid (photo provided by Western Pacific Fisheries, Inc.)

Mobulid sorting grids were modified and successfully implemented for all participating vessels. High levels of fisher‐led customization on vessels resulted in highly varied grid models among vessels, including models with bungee grids and chain suspension systems (Figure 3a), flexible rope grids (Figure 3b), grids with tapered corners (Figure 3c), and raised grids (Figure 3d).

### Mobulid captures

**FIGURE 4:**
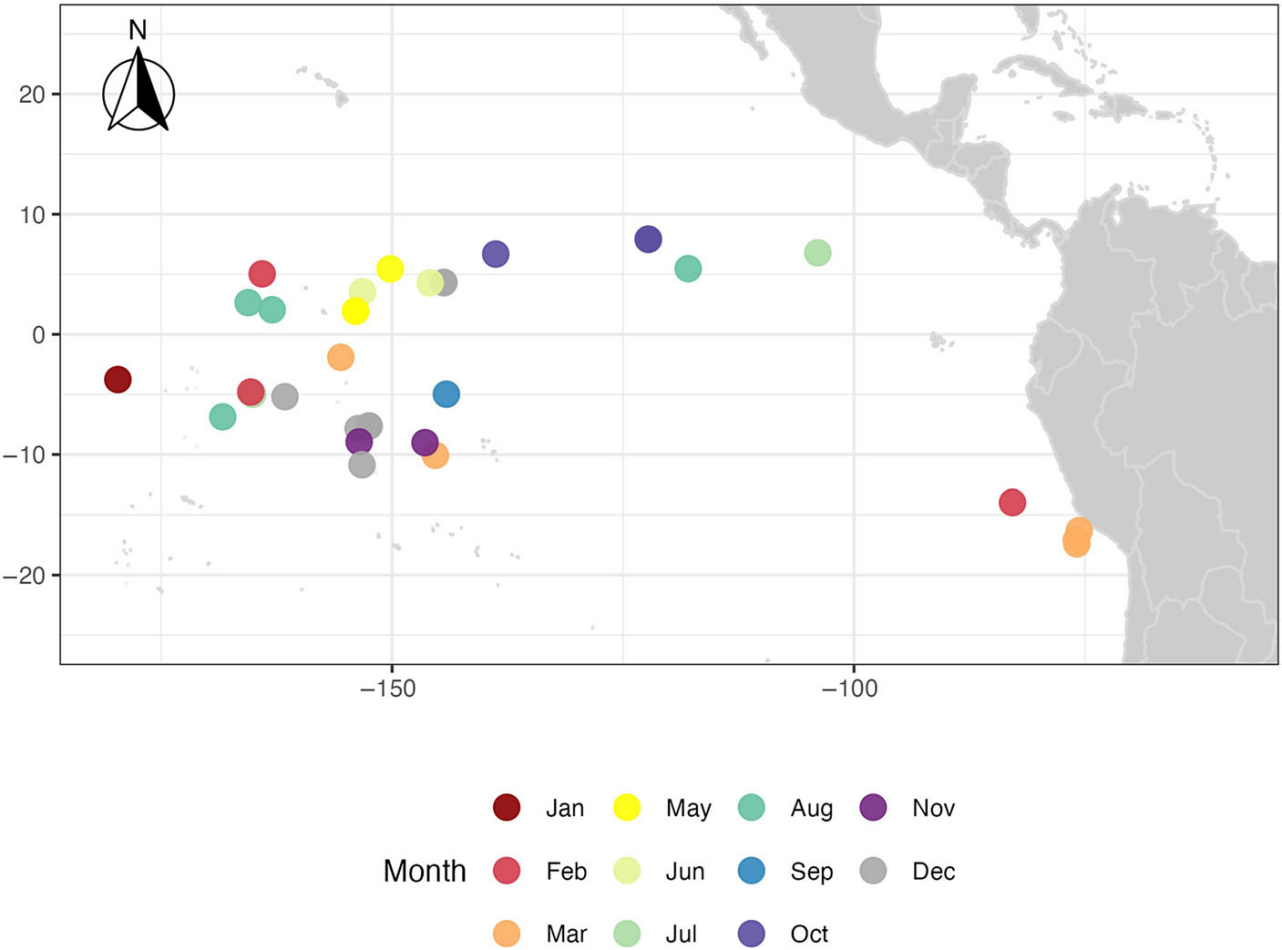
Location and month of mobulid ray captures by tuna purse seine vessels in the Pacific Ocean for which mobulid capture data were collected from 2022 to 2024. No captures occurred in April.

A total of 41 mobulid captures by participating vessels were documented in the eastern and western Pacific Ocean during the study period (Figure 4). The main target species captured in FADs and unassociated sets with mobulids was primarily skipjack (except for one set, which was bigeye tuna). Set size ranged from 0 to 435 t (mean 59.6 t). Half the mobulids appeared before the third brail; however, the average brail number in which a mobulid was encountered was the sixth brail. Capture data were collected primarily by fishing crew (*n* = 37) as opposed to by scientists (*n* = 4).

Most individuals were relatively small (mean disc width [SD] = 191.4 cm [49]). Of the individuals identified to the species level (*n* = 21), *Mobula mobular* was the species most frequently identified (*n* = 6), followed by *Mobula tarapacana* (*n* = 5), *Mobula thurstoni* (*n* = 5), *M. munkiana* (*n* = 3), and *M. birostris* (*n* = 2). On 5 occasions, representing 24% of individuals, mobulids were caught as pairs in the same set or, in one case, in 2 different sets but on the same day.

### Release methods

**FIGURE 5:**
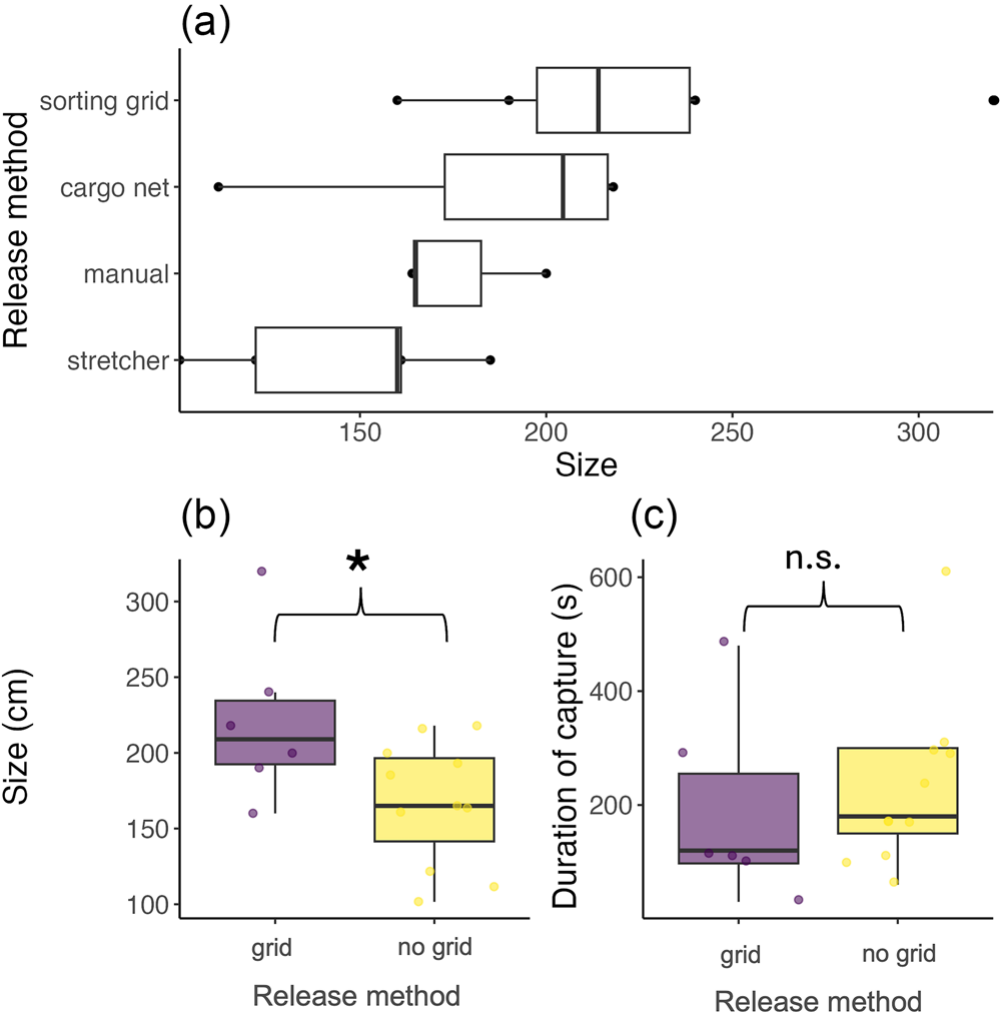
The (a) mobulid release method in relation to the size of individuals, (b) size of the mobulid relative to the release method (sorting grid vs. no sorting grid), and (c) duration of capture relative to the release method (sorting grid vs. no sorting grid) (lines in bars, median; bar length, interquartile range [IQR]; bar ends, 25th percentile and 75th percentile; whiskers, minimum and maximum values within 1.5 × IQR).

Of the 41 individuals captured, release method information was available for 34 individuals. Seven different release methods from the deck were documented. Most individuals were released after reaching the vessel deck with a mobulid sorting grid (*n* = 10), stretcher (*n* = 9), or cargo net (*n* = 6), or they were released manually (*n* = 6) (Figure 5a).

In 3 cases, mobulids were released before reaching the deck. One individual was released from the brailer while fish was in it, one was released from the net during the process of rolling it onto the vessel deck, and one was released directly from the net as it was being gathered at the side of the vessel (sacking operation) before brailing began.

### Mobulid sorting grid performance

After excluding individuals that were released prior to reaching the deck and those for whom release duration or size data were missing, 21 individuals could be included in the evaluation of sorting grid performance. The mean time on deck for these individuals (time from the animal appearing in the brailer to its release) was 3 min 26 s (range 0.5–10.0 min).

Mobulids released using the sorting grid were significantly larger (mean disc width [SD] = 222 cm [44], *n* = 8) than those released using other methods (mean disc width = 173 cm [47], *n* = 13) (*p* < 0.05) (Figure 5b). Despite the larger size of mobulids released using the sorting grid, the mean time on deck for grid releases (3:10; range: 1:30–8:00 min) was not significantly different than the mean time on deck for other release methods (3:52; range: 1:00–10:02 min, *p* = 0.868) (Figure 5c). Despite small sample size, we observed a large effect size (Cohen's *d* = 1.07) for disc width, confirming that the sorting grid significantly aids in the release of large mobulids without increasing handling time.

## DISCUSSION

### Sorting grids are effective for bycatch release

Sorting grids for large mobulids caught by purse seine vessels improved handling efficiency and allowed fishers to return large animals to the sea as quickly as they returned smaller, easier‐to‐handle individuals. There were significant differences in the sizes of animals released using the various methods. The sorting grid was typically used for much larger animals, whereas other manual methods were used for smaller animals. This is because although small animals can be lifted and released by one or 2 crew members, these larger animals typically require multiple crew members to assist with handling. During tuna brailing operations, crew availability is normally limited due to the time‐sensitive nature of transferring tuna, which must be done quickly to prevent histamine buildup. However, we found that release duration did not vary significantly across methods. Although the use of the sorting grid did not reduce overall handling time compared with other methods, it likely prevented prolonged delays (and mobulid mortalities) that would otherwise have occurred when attempting to manually lift large individuals. Prior to the introduction of the grid, large mobulids may have remained on deck longer while awaiting sufficient crew support (Cronin et al., 2022; Murua et al., 2022). This suggests that sorting grids are effective in facilitating the safe and rapid release of larger individuals without imposing additional time burdens during the fishing operation.

Fishers did not always opt to use the sorting grid. We found that for smaller mobulids, fishers generally used other manual methods. This reflects the reality that small individuals can be lifted and released by hand, whereas larger animals require mechanical assistance. This finding highlights the utility of the sorting grid in facilitating the safe release of larger mobulids that cannot be manually handled. Ensuring that grids are available, maintained, and properly used may be especially critical for improving postrelease survival among the largest individuals, which may also be the most vulnerable to handling stress (Stewart et al., 2024).

Recent work by Stewart et al. (2024) indicates that the most important contributing factor to maximize mobulid postrelease survival in tuna purse seiners is reducing release time (i.e., time out of the water), with 3 minutes being a goal to minimize postrealease mortality. We found that the mobulid sorting grid has the potential to facilitate rapid release, especially for large animals within this 3‐minute window. It also has the potential to minimize stress associated with capture, as the animal no longer needs to be manipulated by the gill slits, cephalic fins, or other body parts to pull it out of the brailer. These findings support the utility of sorting grids as a meaningful BRD that can enhance conservation outcomes for large mobulids without compromising operational efficiency.

Currently, purse seine fishers have many years of experience releasing mobulids manually, with stretchers, and using cargo nets, but this was the first time that the U.S. tropical tuna purse seine fishers employed these types of sorting grids. We expect that as fishers gain experience handling mobulids with this new device, they will be more efficient, thus further reducing operational release times and potentially increased postrelease survival. For example, a concurrent study in the Atlantic Ocean with a vessel's crew experienced in using the sorting grid demonstrated even faster release times for large mobulids, with release ranging between 1 and 2 min (Murua et al., 2022).

However, despite the potential utility of the sorting grid, quantitative estimates of the difference in postrelease mortality rates between handling methods would be useful. Future research should focus on using satellite tags to measure postrelease mortality with and without the use of the sorting grid to assess its efficacy. Fortunately, further evaluation of the sorting grids with satellite tags is planned for additional tuna purse seine vessels, including smaller vessels, in the eastern Pacific, which will help fill this knowledge gap (IATTC, 2020).

Given the rarity of mobulids in fishing sets and thus the relatively low sample sizes reflected here, future efforts should also seek to target vessels fishing in areas and seasons of relatively higher mobulid bycatch, like coastal areas off Peru, the Galapagos, and the Costa Rica thermal dome, which will allow further testing and refinement of the sorting grid (Lezama‐Ochoa et al., 2019). When planning data collection for this study, we conducted an exploration of mobulid bycatch across different regions in the Pacific Ocean and consulted scientific staff working for tRFMOs. However, predicting mobulid interactions with purse seine vessels is difficult, and even fishers found it difficult to identify the optimal spatial and temporal strata for our cruises. This lack of knowledge regarding mobulid bycatch in tRFMOs, particularly for vessels without observers (e.g., smaller purse seine vessels), highlights the need for further data collection by onboard observers or electronic monitoring systems.

Interestingly, our evaluation of a bycatch reduction device also highlighted precapture release. In 3 cases in this study, individuals were released before reaching the vessel deck, either directly from the net while it was being hauled in or during the sacking‐up phase (i.e., the final stage of hauling where fish are concentrated in a small area next to the vessel for brailing), or from the brailer. Although these methods may represent an ideal release scenario with minimal handling, anecdotal evidence from fishers (Cronin et al., 2022) suggests that such releases are both rare and operationally difficult. Typically, mobulids dive to the bottom of the net during capture, making them difficult to detect until later in the process, often after most of the tuna have already been removed (Stewart et al., 2024). As such, predeck release opportunities are unpredictable and may not always be feasible. However, the fact that these kinds of releases occurred unprompted on multiple occasions suggests a critical area for future research: identifying the enabling conditions for successful predeck release. It is possible that this behavior is conducted by a few highly skilled operators or on vessels with better equipment or operational characteristics to facilitate this maneuver; interviewing these crew members could be key to understanding whether the behavior could be replicated.

Although we did not quantify operational impacts such as set or brail duration (e.g., time it takes to bring tuna onboard), informal crew feedback suggested that the sorting grid did not significantly disrupt normal fishing activities. Future research could explore whether grid use introduces trade‐offs in fishing efficiency. Additionally, although our analysis focused on mobulids, sorting grids may have broader conservation benefits for other large bycatch species that cannot be easily lifted. However, limited observations of other large bycatch species during this study prevented us from evaluating these effects. Further research is needed to assess the grid's performance across a wider range of taxa.

### Improving data collection for mobulids

Our results suggest that cooperation with fishing crew is essential for obtaining data on rare bycatch; in fact, only 4 of 41 captures were recorded by biologists over the course of a combined 6 months at sea. However, a limitation of this study was that incomplete data by fishing crews required the exclusion of nearly half of the captured individuals from our analysis of sorting grid performance (21 out of 41 total captures). Although fisher‐led data collection greatly increased sampling, it can also lead to variability in data quality. These gaps point to the need for scientists to employ clearer training, simplified protocols, and possibly digital tools to help crews consistently record release conditions. Still, these challenges should not discourage future collaboration between scientists and fishers. Given the high cost and logistical complexity of placing researchers on tuna vessels, partnering with fishing crews remains one of the most effective ways to collect ocean‐wide data on rare bycatch species. Future studies should invest more time in codeveloping easy‐to‐use protocols with fishing crews to improve both the accuracy and feasibility of at‐sea data collection. In addition to better training, research in partnership with fishing crews might explore the use of incentives like financial compensation and increased recognition to motivate consistent, high‐quality data collection by fishers.

Although we reported species‐level identifications of mobulids, these should be interpreted cautiously. Mobulids appear morphologically similar, and species identification is a persistent challenge. One example of these issues was the identification by fishing crew in this study of *M. munkiana* individuals on the high seas, outside of the species’ known coastal distribution range (Notarbartolo‐Di‐Sciara, 1988). This suggests that some identification errors may have occurred, potentially due to limitations in training or field experience among fishing crew data collectors. This is a potential barrier to evaluating the efficacy of the sorting grid, as survival is likely highly variable among mobulid species (Stewart et al., 2024). Improving species identification skills is essential to enhancing the accuracy of data collection. Due to the similar appearance of mobulid ray species, training workshops, standardized identification protocols, and the use of newly developed species identification guides with clear and easy‐to‐follow keys could help improve fisher and observer species identification skills (Hutchinson et al., 2023). These efforts could be complemented by the implementation of artificial intelligence‐based smart species identification tools and genetic studies that can validate observer and fisher species identification (IATTC, 2024b, 2024c). The use of electronic monitoring systems, combined with artificial intelligence for data processing, could significantly enhance future data collection on mobulid species as well (van Helmond et al., 2020). These technologies would provide more accurate and comprehensive information on species‐specific interactions with tropical tuna purse seine fleets, ensuring more consistent and detailed data collection across regions and timeframes.

### Fisher customization and further adoption of sorting grids

It has been widely documented that BRDs that involve fisher participation are more likely to be successful (Barz et al., 2020; Jenkins, 2023; Murua et al., 2022; Suuronen, 2022). We attempted to include fisher expertise, perspectives, and lived experiences by eliciting feedback throughout the process and allowing them to fabricate and customize the mobulid sorting grids tailored to the unique operational constraints of each vessel. Construction of the sorting grid was feasible for the fleet due to the availability of low‐cost materials already in use on the vessels. Reported feedback from the crew reflected that the use of the mobulid sorting grids in most cases seemed to work well. Further, one explanation for the difference in sizes for animals released with the mobulid sorting grid versus other methods is that fishers choose to use the grid for larger animals because they know that otherwise, it will be harder and take longer to release the individual. This is promising, given the need to align bycatch handling recommendations with behavioral incentives to achieve conservation goals (Suuronen, 2022). Incorporating fishers in the development and deployment of these technologies can ensure that they are not only effective for bycatch mortality reduction but also more easily implemented in the fishery. Further, the direct involvement of fishers from the start in the design, construction, and trials of the sorting grids can promote a sense of ownership and stewardship for this technology.

Although our study focused on large‐scale industrial purse seine fisheries, the sorting grid concept may have broader applicability in smaller purse seiners, where large bycatch species such as mobulids might be encountered. In any case, successful implementation in these contexts may require improved monitoring strategies, for example, use of electronic monitoring systems (van Helmond et al., 2020). Fortunately, given the low cost and technical simplicity of the device, it could be feasible and practical even in low‐income settings where bycatch mitigation is otherwise difficult.

### Management recommendations

Our results demonstrate the potential utility of sorting grids to enhance mobulid bycatch mitigation efforts in tuna purse seine fisheries. Broader implementation of these devices supported by tRFMOs could significantly contribute to the conservation of these vulnerable species. Measures that dictate recommended and required practices for mobulids captured by tuna fisheries are set by tRFMOs. Beginning in 2015, all of the 4 major tropical tRFMOs began to adopt measures that prohibit the retention, transshipment, or landing of mobulids caught in their Convention Area (the region where the tRFMO is responsible for managing fisheries). These measures also require prompt release of bycaught individuals; however, they do not specify how to rapidly release large individuals who cannot be released manually (Cronin, et al., 2022). Given these measures and the rapid release times documented here for large individuals, tRFMOs could consider updating best practices and conservation and management measures to require fishers to use mobulid sorting grids on their vessels. For example, the use of mobulid sorting grids has been recommended by the Indian Ocean Tuna Commission though not yet required (IOTC, 2024). Countries could also use this device to make meaningful progress toward their own conservation goals. For example, in the United States, this approach could help achieve conservation targets for oceanic manta rays, which are listed under the federal Endangered Species Act (NOAA, 2018). It could also help vessels and fishery sectors achieve bycatch mitigation goals set by fishery improvement projects and other sustainability initiatives. These efforts can play an important role in ensuring the long‐term protection and recovery of threatened mobulid populations.

## AUTHOR CONTRIBUTIONS

Melissa R. Cronin, Gala Moreno, and Jefferson Murua conceived of the study. Gala Moreno and Victor Restrepo obtained funding for the study, and Kelly M. Zilliacus contributed to the administration of funds. Gala Moreno and Jefferson Murua led fisher workshops and vessel site visits to guide fishers in the deployment of the mobulid sorting grids on vessels. Melissa R. Cronin collected and analyzed the data sorting grid performance data. Melissa R. Cronin, Gala Moreno, and Jefferson Murua wrote and edited the first draft. Donald A. Croll, Melanie Hutchinson, Nerea Lezama‐Ochoa, Jon Lopez, Hilario Murua, Marta D. Palacios, Victor Restrepo, Joshua D. Stewart, Yonat Swimmer, and Kelly M. Zilliacus edited and revised subsequent drafts.

## Supporting information



Supporting Information

Supporting Information

## References

[cobi70150-bib-0001] Alfaro‐Cordova, E. , Del Solar, A. , Alfaro‐Shigueto, J. , Mangel, J. C. , Diaz, B. , Carrillo, O. , & Sarmiento, D. (2017). Captures of manta and devil rays by small‐scale gillnet fisheries in northern Peru. Fisheries Research, 195, 28–36.

[cobi70150-bib-0002] Barz, F. , Eckardt, J. , Meyer, S. , Kraak, S. B. M. , & Strehlow, H. V. (2020). ‘Boats don't fish, people do’‐ How fishers′ agency can inform fisheries‐management on bycatch mitigation of marine mammals and sea birds. Marine Policy, 122, Article 104268.

[cobi70150-bib-0043] Convention on International Trade in Endangered Species of Wild Fauna and Flora (CITES) . (2025). CITES appendices. https://cites.org/eng/app/appendices.php 10.1159/000459796 712806

[cobi70150-bib-0003] Couturier, L. I. E. , Marshall, A. D. , Jaine, F. R. A. , Kashiwagi, T. , Pierce, S. J. , Townsend, K. A. , Weeks, S. J. , Bennett, M. B. , & Richardson, A. J. (2012). Biology, ecology and conservation of the Mobulidae. Journal of Fish Biology, 80, 1075–1119. 22497374 10.1111/j.1095-8649.2012.03264.x

[cobi70150-bib-0004] Croll, D. A. , Dewar, H. , Dulvy, N. K. , Fernando, D. , Francis, M. P. , Galván‐Magaña, F. , Hall, M. , Heinrichs, S. , Marshall, A. , Mccauley, D. , Newton, K. M. , Notarbartolo‐Di‐Sciara, G. , O'Malley, M. , O'Sullivan, J. , Poortvliet, M. , Roman, M. , Stevens, G. , Tershy, B. R. , & White, W. T. (2016). Vulnerabilities and fisheries impacts: The uncertain future of manta and devil rays. Aquatic Conservation: Marine and Freshwater Ecosystems, 26, 562–575.

[cobi70150-bib-0005] Cronin, M. R. , Amaral, J. E. , Jackson, A. M. , Jacquet, J. , Seto, K. L. , & Croll, D. A. (2022). Policy and transparency gaps for oceanic shark and rays in high seas tuna fisheries. Fish and Fisheries, 24, 56–70.

[cobi70150-bib-0006] Cronin, M. R. , Croll, D. A. , Hall, M. A. , Lezama‐Ochoa, N. , Lopez, J. , Murua, H. , Murua, J. , Restrepo, V. , Rojas‐Perea, S. , Stewart, J. D. , Waldo, J. L. , & Moreno, G. (2022). Harnessing stakeholder knowledge for the collaborative development of Mobulid bycatch mitigation strategies in tuna fisheries. ICES Journal of Marine Science, 80(3), 1–15.

[cobi70150-bib-0047] Deakos, M. H. (2012). The reproductive ecology of resident manta rays (Manta alfredi) off Maui, Hawaii, with an emphasis on body size. Environmental Biology of Fishes, 94, 443–456.

[cobi70150-bib-0007] Dulvy, N. K. , Baum, J. K. , Clarke, S. , Compagno, L. J. V. , Cortés, E. , Domingo, A. , Fordham, S. , Fowler, S. , Francis, M. P. , Gibson, C. , Martínez, J. , Musick, J. A. , Soldo, A. , Stevens, J. D. , & Valenti, S. (2008). You can swim but you can't hide: The global status and conservation of oceanic pelagic sharks and rays. Aquatic Conservation: Marine and Freshwater Ecosystems, 18, 459–482.

[cobi70150-bib-0008] Dulvy, N. K. , Pacoureau, N. , Rigby, C. L. , Pollom, R. A. , Jabado, R. W. , Ebert, D. A. , Finucci, B. , Pollock, C. M. , Cheok, J. , Derrick, D. H. , Herman, K. B. , Sherman, C. S. , VanderWright, W. J. , Lawson, J. M. , Walls, R. H. L. , Carlson, J. K. , Charvet, P. , Bineesh, K. K. , Fernando, D. , … Simpfendorfer, C. A. (2021). Overfishing drives over one‐third of all sharks and rays toward a global extinction crisis. Current Biology, 31, 4773.e8–4787.e8. 34492229 10.1016/j.cub.2021.08.062

[cobi70150-bib-0009] Dulvy, N. K. , & Simpfendorfer, C. A. (2022). Guiding random acts of kindness: Conservation planning for sharks and rays. In J. Carrier , C. A. Simpfendorfer , M. R. Heithaus , and K. A. Yopak (Eds.), Biology of sharks and their relatives (pp. 715–736). CRC Press.

[cobi70150-bib-0010] Fernando, D. , & Stewart, J. D. (2021). High bycatch rates of manta and devil rays in the “small‐scale” artisanal fisheries of Sri Lanka. PeerJ, 9, Article e11994. 34589295 10.7717/peerj.11994 PMC8434810

[cobi70150-bib-0011] Griffiths, S. P. , & Lezama‐Ochoa, N. (2021). A 40‐year chronology of the vulnerability of spinetail devil ray (*Mobula mobular*) to eastern Pacific tuna fisheries and options for future conservation and management. Aquatic Conservation: Marine and Freshwater Ecosystems, 31, 2910–2925.

[cobi70150-bib-0012] Hall, M. , & Roman, M. (2013). Bycatch and non‐tuna catch in the tropical tuna purse seine fisheries of the world (Fisheries and Aquaculture Technical Paper No. 568). FAO.

[cobi70150-bib-0036] van Helmond, A. T. M. , Mortensen, L. O. , Plet‐Hansen, K. S. , Ulrich, C. , Needle, C. L. , Oesterwind, D. , Kindt‐Larsen, L. , Catchpole, T. , Mangi, S. , Zimmermann, C. , Olesen, H. J. , Bailey, N. , Bergsson, H. , Dalskov, J. , Elson, J. , Hosken, M. , Peterson, L. , McElderry, H. , Ruiz, J. , … Poos, J. J. (2020). Electronic monitoring in fisheries: Lessons from global experiences and future opportunities. Fish and Fisheries, 21, 162–189.

[cobi70150-bib-0013] Hutchinson, M. , Justel‐Rubio, A. , & Restrepo, V. (2020). At‐sea tests of releasing sharks from the net of a tuna purse seiner in the Atlantic ocean. Collective Volume of Scientific Papers, ICCAT, 76(9), 61–72.

[cobi70150-bib-0014] Hutchinson, M. , Lopez, J. , Wiley, B. , Pulvenis, J.‐F. , Altamirano, E. , & Aires‐da‐Silva, A. (2023). Knowledge and research gaps to the implementation of best handling and release practices for vulnerable species (EB‐01‐01 REV). Working Group on Ecosystem & Bycatch, IATTC.

[cobi70150-bib-0015] Hutchinson, M. , Poisson, F. , & Swimmer, Y. (2017). Developing best handling practice guidelines to safely release mantas, mobulids and stingrays captured in commercial fisheries (PIFSC Working Paper WP‐17‐006). National Oceanic and Atmospheric Administration.

[cobi70150-bib-0046] Indian Ocean Tuna Commission (IOTC) . (2024). Report of the 27th Session of the IOTC Scientific Committee . Cape Town, 2 –6 December 2024. IOTC–2024–SC27–R[E].

[cobi70150-bib-0016] Inter‐American Tropical Tuna Commission (IATTC) . (2020). Project M.1.b Test sorting grids .

[cobi70150-bib-0017] Inter‐American Tropical Tuna Commission (IATTC) . (2024a). DOCUMENT SAC‐15‐01 CORR. The Tuna Fishery in the Eastern Pacific Ocean in 2023 .

[cobi70150-bib-0018] Inter‐American Tropical Tuna Commission (IATTC) . (2024b). DOCUMENT SAC‐15 INF‐E.a, Project B1a (Scientific Advisory Committee) .

[cobi70150-bib-0039] Inter‐American Tropical Tuna Commission (IATTC) . (2024c). Improving smart species identification tools. IATTC. https://www.iattc.org/en‐US/Research/Project/Detail/B‐1‐a

[cobi70150-bib-0042] International Union for Conservation of Nature (IUCN) . (2025). The IUCN Red List of Threatened Species. Version 2025‐1. https://www.iucnredlist.org

[cobi70150-bib-0019] Jenkins, L. D. (2023). Turtles, TEDs, tuna, dolphins, and diffusion of innovations: Key drivers of adoption of bycatch reduction devices. ICES Journal of Marine Science, 80(3), 417–436.

[cobi70150-bib-0044] Justel‐Rubio, A. (2024). A Snapshot of the Large‐Scale Tropical Tuna Purse Seine Fishing Fleets as of June 2024 (Version 12). ISSF Technical Report 2024‐05. International Seafood Sustainability Foundation.

[cobi70150-bib-0048] Komoroske, L. M. , & Lewison, R. L. (2015). Addressing fisheries bycatch in a changing world. Frontiers in Marine Science, 2, 83. 10.3389/fmars.2015.00083

[cobi70150-bib-0052] Lewison, R. L. , Crowder, L. B. , Wallace, B. P. , Moore, J. E. , Cox, T. , Zydelis, R. , McDonald, S. , DiMatteo, A. , Dunn, D. C. , Kot, C. Y. , Bjorkland, R. , Kelez, S. , Soykan, C. , Stewart, K. R. , Sims, M. , Boustany, A. , Read, A. J. , Halpin, P. , Nichols, W. J. , & Safina, C. (2014). Global patterns of marine mammal, seabird, and sea turtle bycatch reveal taxa‐specific and cumulative megafauna hotspots. Proceedings of the National Academy of Sciences of the United States of America, 111, 5271–5276. 24639512 10.1073/pnas.1318960111 PMC3986184

[cobi70150-bib-0020] Lezama‐Ochoa, N. , Hall, M. , Román, M. , & Vogel, N. (2019). Spatial and temporal distribution of mobulid ray species in the eastern Pacific Ocean ascertained from observer data from the tropical tuna purse‐seine fishery. Environmental Biology of Fishes, 102, 1–17.

[cobi70150-bib-0051] Marshall, A. D. , & Bennett, M. B. (2010). Reproductive ecology of the reef manta ray Manta alfredi in southern Mozambique. Journal of Fish Biology, 77, 169–190. 20646146 10.1111/j.1095-8649.2010.02669.x

[cobi70150-bib-0021] Moazzam, M. (2018). Unprecedented decline in the catches of mobulids: An important component of tuna gillnet fisheries of the Northern Arabian Sea (IOTC‐2018‐WPEB14‐30). WWF Pakistan.

[cobi70150-bib-0022] Murua, H. , Zudaire, I. , Tolotti, M. , Murua, J. , Capello, M. , Basurko, O. C. , Krug, I. , Grande, M. , Arregui, I. , Uranga, J. , Ferarios, J. M. , Sabarros, P. , Ruiz, J. , Baidai, Y. , Ramos, M. L. , Báez, J. C. , Abascal, F. , Arrizabalaga, H. , Moreno, G. , … Santiago, J. (2023). Lessons learnt from the first large‐scale biodegradable FAD research experiment to mitigate drifting FADs impacts on the ecosystem. Marine Policy, 148, Article 105394.

[cobi70150-bib-0023] Murua, J. , Ferarios, J. M. , Grande, M. , Onandia, I. , Moreno, G. , Murua, H. , & Santiago, J. (2022). Developing bycatch reduction devices in tropical tuna purse seine fisheries to improve elasmobranch release (SCRS/2022/108). Inter‐American Tropical Tuna Commission.

[cobi70150-bib-0024] Murua, J. , Ferarios, J. M. , Grande, M. , Ruiz, J. , Cuevas, N. , Krug, I. , Onandia, I. , Zudaire, I. , Salgado, A. , Erauskin‐Extramiana, M. , Lopetegui‐Eguren, L. , & Santiago, J. (2024). Incorporating bycatch release devices in guidelines for best bycatch handling and release practices in tropical tuna purse seiners. Inter‐American Tropical Tuna Commission.

[cobi70150-bib-0025] Murua, J. , Ferarios, J. M. , Moreno, G. , Grande, M. , & Murua, H. (2023). ISSF workshop on deck bycatch release devices (BRDs) for vulnerable species in tropical tuna purse seiners (ISSF technical report 2023–11A). International Seafood Sustainability Foundation.

[cobi70150-bib-0026] Murua, J. , Ferarios, J. M. , Moreno, G. , Onandia, I. , Ruiz, J. , Zudaire, I. , Santiago, J. , Murua, H. , & Restrepo, V. (2021). Developing solutions to increase survival rates of vulnerable bycatch species in tuna purse seiner FAD fisheries (IOTC‐2021‐WGFAD02‐11_rev1). Indian Ocean Turn Commission.

[cobi70150-bib-0027] Murua, J. , Moreno, G. , Dagorn, L. , Itano, D. , Hall, M. , Murua, H. , & Restrepo, V. (2023). Improving sustainable practices in tuna purse seine fish aggregating device (FAD) fisheries worldwide through continued collaboration with fishers. Frontiers in Marine Science, 10, Article 1074340.

[cobi70150-bib-0028] National Oceanic and Atmospheric Administration (NOAA) . (2024). High Seas Western and Central Pacific Ocean Tuna Purse Seine Fishery—MMPA list of fisheries . Author.

[cobi70150-bib-0040] National Oceanic and Atmospheric Administration (NOAA) . (2018). Final Rule To List the Giant Manta Ray as Threatened Under the Endangered Species Act. 50 CFR Part 223 [Docket No. 160105011‐7999‐03] RIN 0648‐XE390. https://www.federalregister.gov/documents/2018/01/22/2018‐01031/endangered‐and‐threatened‐wildlife‐and‐plants‐final‐rule‐to‐list‐the‐giant‐manta‐ray‐as‐threatened

[cobi70150-bib-0029] Notarbartolo‐Di‐Sciara, G. (1988). Natural history of the rays of the genus *Mobula* in the Gulf of California. Fishery Bulletin, 86(1), 45–66.

[cobi70150-bib-0030] Pardo, S. A. , Kindsvater, H. K. , Cuevas‐Zimbrón, E. , Sosa‐Nishizaki, O. , Pérez‐Jiménez, J. C. , & Dulvy, N. K. (2016). Growth, productivity and relative extinction risk of a data‐sparse devil ray. Scientific Reports, 6, Article 33745. 27658342 10.1038/srep33745 PMC5034314

[cobi70150-bib-0049] Peatman, T. (2021). Bycatch Estimates—Tables 6–9, SC17‐ST‐IP‐06 (Updated PS bycatch estimates in the WCPO). WCPFC. https://www.wcpfc.int/doc/bycatch‐estimates‐xlsx

[cobi70150-bib-0045] Poisson, F. , Séret, B. , Vernet, A.‐L. , Goujon, M. , & Dagorn, L. (2014). Collaborative research: Development of a manual on elasmobranch handling and release best practices in tropical tuna purse‐seine fisheries. Marine Policy, 44, 312–320.

[cobi70150-bib-0031] Rohner, C. , Flam, A. , Pierce, S. , & Marshall, A. (2017). Steep declines in sightings of manta rays and devilrays (Mobulidae) in southern Mozambique. *PeerJ* . https://peerj.com/preprints/3051.pdf

[cobi70150-bib-0032] Stevens, G. , Fernando, D. , Dando, M. , & di Sciara, G. N. (2018). Guide to the manta and devil rays of the world. Princeton University Press.

[cobi70150-bib-0050] Stevens, G. M. W. (2016). Conservation and Population Ecology of Manta Rays in the Maldives. Phd. University of York. https://etheses.whiterose.ac.uk/16981/

[cobi70150-bib-0033] Stewart, J. D. , Cronin, M. R. , Largacha, E. , Lezama‐Ochoa, N. , Lopez, J. , Hall, M. , Hutchinson, M. , Jones, E. G. , Francis, M. , Grande, M. , Murua, J. , Rojo, V. , & Jorgensen, S. J. (2024). Get them off the deck: Straightforward interventions increase post‐release survival rates of manta and devil rays in tuna purse seine fisheries. Biological Conservation, 299, Article 110794.

[cobi70150-bib-0034] Stewart, J. D. , Jaine, F. R. A. , Armstrong, A. J. , Armstrong, A. O. , Bennett, M. B. , Burgess, K. B. , Couturier, L. I. E. , Croll, D. A. , Cronin, M. R. , Deakos, M. H. , Dudgeon, C. L. , Fernando, D. , Froman, N. , Germanov, E. S. , Hall, M. A. , Hinojosa‐Alvarez, S. , Hosegood, J. E. , Kashiwagi, T. , Laglbauer, B. J. L. , … Stevens, G. M. W. (2018). Research priorities to support effective manta and devil ray conservation. Frontiers in Marine Science, 5, Article 314.

[cobi70150-bib-0035] Suuronen, P. (2022). Understanding perspectives and barriers that affect fishers’ responses to bycatch reduction technologies. ICES Journal of Marine Science, 79, 1015–1023.

[cobi70150-bib-0037] Venables, S. K. , Rohner, C. A. , Flam, A. L. , Pierce, S. J. , & Marshall, A. D. (2024). Persistent declines in sightings of manta and devil rays (Mobulidae) at a global hotspot in southern Mozambique. Environmental Biology of Fishes, 108, 749–765.

[cobi70150-bib-0038] Ward‐Paige, C. A. , Davis, B. , & Worm, B. (2013). Global population trends and human use patterns of Manta and Mobula rays. PLoS ONE, 8, Article e74835. 24040348 10.1371/journal.pone.0074835 PMC3770565

