# Supplementary material for: Evidence for a fisher‐designed solution to manta and devil ray bycatch in tuna fisheries
Source: Conserv Biol. 2025 Oct 22;40(1):e70150. doi: 10.1111/cobi.70150 (PMC12856801; doi:10.1111/cobi.70150)

Appendix: Manta Sorting Grid Construction Instructions for Purse Seine Vessels

Prepared by Melissa Cronin (Mobula Conservation, University of California, Santa Cruz), Jefferson Murua (AZTI), and Gala Moreno (International Seafood Sustainability Foundation)

Mobulid rays (manta and devil rays), are highly vulnerable species. Tropical tuna purse seine vessels incidentally catch thousands of mobulids each year globally, and survival after release depends heavily on reducing handling time and method [1]. Research has shown that release within three minutes of capture dramatically improves post-release survival [2]. Traditional handling methods on deck often involved physically grasping mobulids by their gill slits or cephalic lobes, which can cause serious injury, stress, and mortality [3]. Larger animals were sometimes lifted by passing a hook or rope through the gills or body and hoisting them with the crane [4]. Many of these harmful handling and release methods are now prohibited by all four tropical tuna Regional Fisheries Management Organizations (RFMOs): IATTC, C-15-04; ICCAT 23-14; IOTC 19/03; WCPFC, CMM 19-05.

The manta sorting grid (**Figure 1)** is designed to eliminate the need for direct manual handling, protect sensitive body parts, and streamline the release process [5]. It allows tuna to pass through while retaining mobulids on top for immediate crane-assisted release overboard—minimizing crew effort, reducing injury risk, and keeping handling time under three minutes. This document contains instructions on how to construct mobulid sorting grids and variations on design that may be used by tuna purse seine vessel crew to construct their own manta sorting grids.

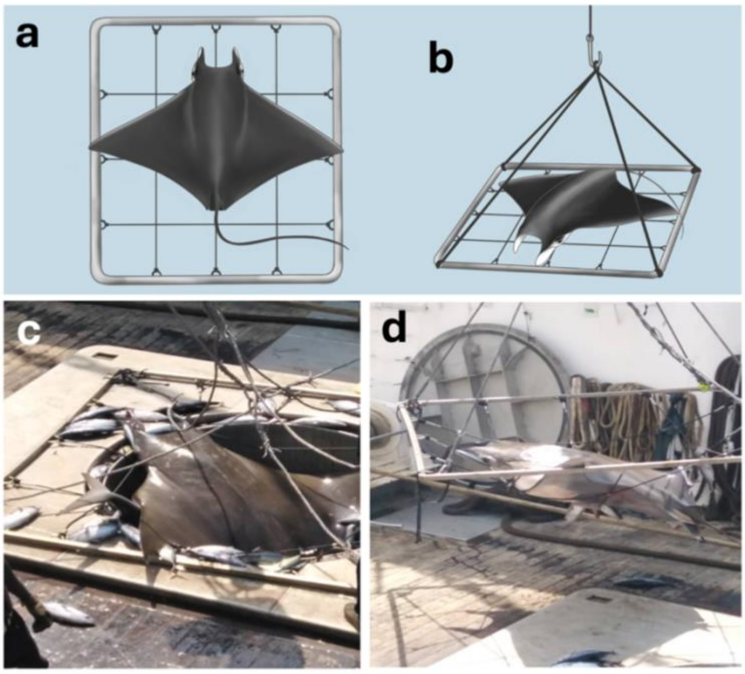

Figure 1. (a) The manta sorting grid allows for rapid release of manta and devil (mobulid) rays. The manta sorting grid should: (b) allow the ray to lie flat, (c) for target fish to pass through the grid lattice, and (d) allow for lifting with a crane using four attached ropes. Illustrations: Life Science Studios; Photos: AZTI

Manta Sorting Grid Construction Instructions

Purpose: The manta sorting grid is a rigid frame with a rope lattice that allows tunas to pass through while retaining medium–large mobulid rays on top for quick release **(Figure 2).** Instructions here are given for a square grid; however, rectangular or circular grids may be used depending on vessel specifications (i.e. the presence or absence of a hopper, the size of the unloading hatch). *See examples of other grid shapes below.*

If the vessel is equipped with a hopper **(Figure 3)**, the grid should be placed inside the hopper's tray, where the entire catch is discharged. In the absence of a hopper, the grid shall be positioned directly over the unloading hatch.

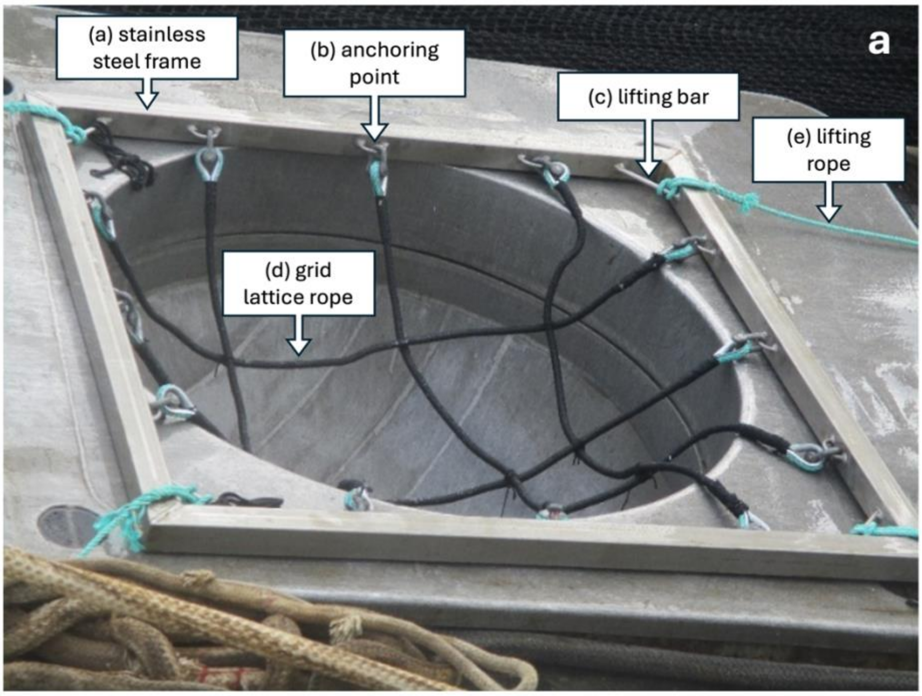

Figure 2. (a) Main components of the manta sorting grid. Photo: AZTI

Materials:

- Stainless steel tubing for the frame (e.g., 200 cm length × 6 cm width × 6 cm height; wall thickness 3–4 mm if hollow)
- Stainless steel half-rings to fasten the ropes, forming the lattice (3–4 per side)
- Solid stainless steel bars for corner lifting points (4 total)
- Durable rope for grid lattice
- Thinner rope for tying crossing points and release loops
- Four equal-length lifting ropes or chains for crane attachment

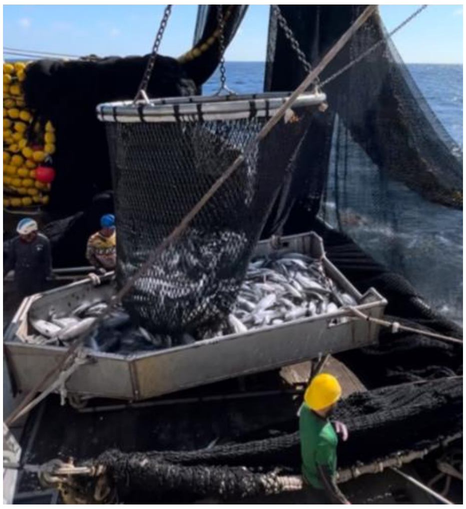

Figure 3. Some vessels use a hopper to discharge the catch and perform preliminary sorting before the fish pass through the unloading hatch into the fish wells. The use of a hopper facilitates improved release of non-target species from the upper deck. Manta sorting grids are compatible with hoppers. Photo: Melissa Cronin, Pacific Princess

Construction Steps:

1. **Measure size:** Take careful measurements of the unloading hatch **(Figure 4a)** or hopper tray **(Figure 3, Figure 4b),** depending on where the brail is emptied, to construct a sorting grid with the correct size.
   - **Unloading hatch use:** frame must be larger than the opening to rest on the vessel deck **(Figure 4b).**
   - **Hopper use:** frame must be narrower than hopper base to fit inside **(Figure 4c)**

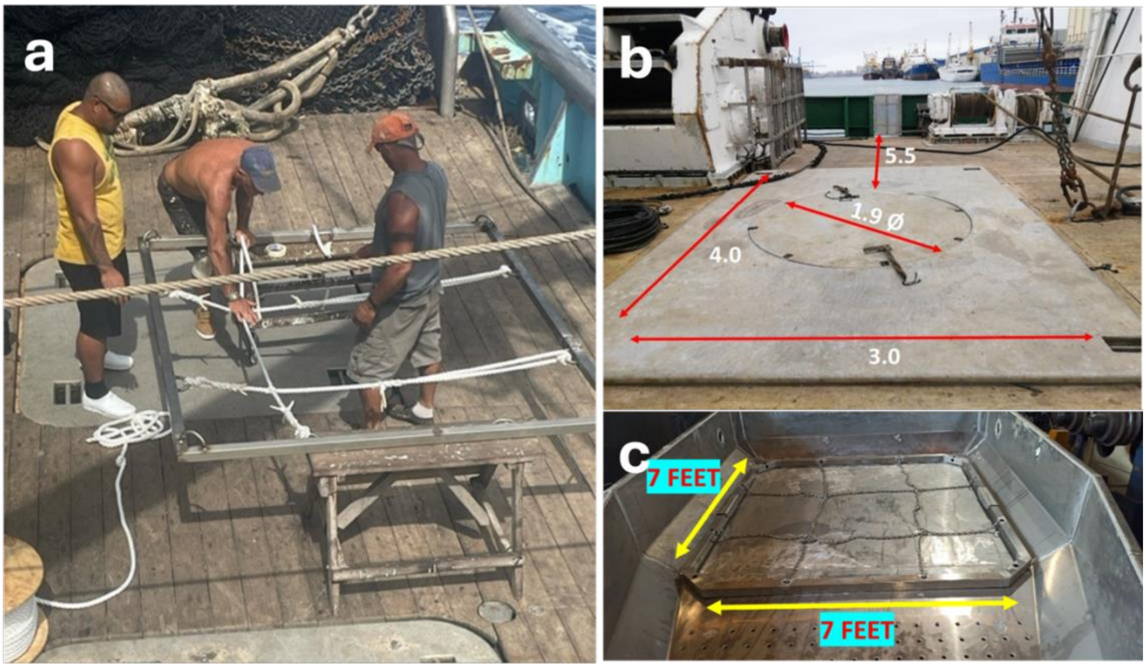

Figure 4. (a) Crew members constructing a manta sorting grid. (b) Unloading hatch measurements and (c) hopper measurements to inform the size of the manta sorting grid. Photos: ISSF, De Silva Sea Encounter Corp., AZTI

2. **Build the frame (Figure 2a):** Use four stainless steel tubes strong enough to hold one or more adult mobulids to create a square grid (see variations on this shape below).
   - Typical measurements for a square grid: 200 cm length x 6 cm wide x 6 cm height (78 inches x 2.5 inches x 2.5 inches)
   - If the stainless-steel tube is hollow, it should have a minimum wall of 4 mm (0.16 inches)

3. **Add anchoring points (Figure 2b):** Weld 3–4 stainless steel half-rings along the inner side of each frame edge, spaced ~25 cm (10 inches) apart. This will provide attachment points for the ropes that will form the grid cells.
   - Alternatives to anchoring points are carabiners or eyebolts

4. **Add lifting bars (Figure 2c):** Weld a solid bar across each inner corner of the frame that will allow for four ropes to be attached for lifting with the crane.

5. **Install grid lattice (Figure 2d):** Tie or attach ropes from each anchoring point to the opposite side of the square. Each rope should connect from one anchoring point to the one on the opposite side of the frame. This will result in a series of squares forming a grid.
   - Grid openings should be large enough for tunas to pass but small enough to retain mobulids
   - Each rope should be slightly longer than the distance between the anchoring points but not so long it hangs too loosely.

6. **Prepare crane lifting system (Figure 2e):** Connect four equal length lifting ropes or chains from each corner bar to the crane hook. On the two starboard-side lifting points, insert thinner rope loops between the frame and heavy lifting lines—these loops will be released to tilt the grid to allow the mobulid to fall into the water.

Step-by-step use on deck:

1. Position the grid over the unloading hatch or inside the hopper tray before brailing begins **(Figure 5, Figure S1).**
2. Empty the brail contents onto the grid; tuna will fall through, while mobulids remain on top **(Figure 5a).**
3. Attach the grid's lifting ropes/chains to the deck crane **(Figure 5b).**
4. Swing the grid to the starboard side **(Figure 5c).**
5. Release the two thinner lifting ropes to allow the mobulid to slide back into the water **(Figure 5d).**
6. Return the grid to position for the next set.

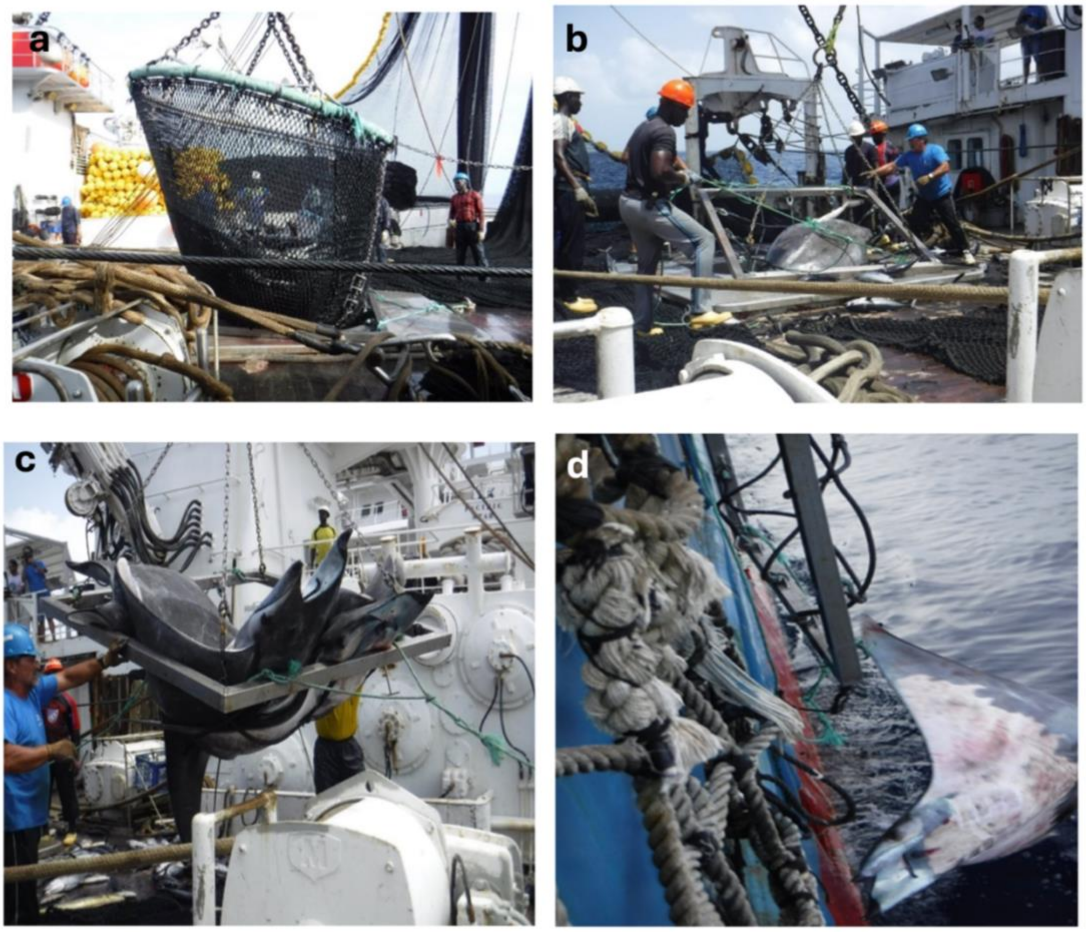

Figure 5. Main steps of the manta ray sorting grid. Photos: AZTI

Vessel-specific adaptations and storage

Vessels are encouraged to design their manta sorting grid using dimensions and rope configurations adapted to each vessel's deck layout and brailing operation. While most manta sorting grids are square, alternative sorting grids have been prepared to fit in the unloading hatch rim in circular shapes **(Figure 6a, Figure 6b).** Grid size may also vary depending on the size of the hatch or hopper **(Figure 6c).** Some grid designs have a hinge in the middle of two of the stainless steel frame pieces, allowing them to fold in half for storage **(Figure 6d).**

If the grid is placed on top of the hopper, it may be fitted with legs to elevate it, creating a gap between the grid and the hopper **(Figure 7).** This will allow tuna to pass more easily through the grid while keeping the rays on top.

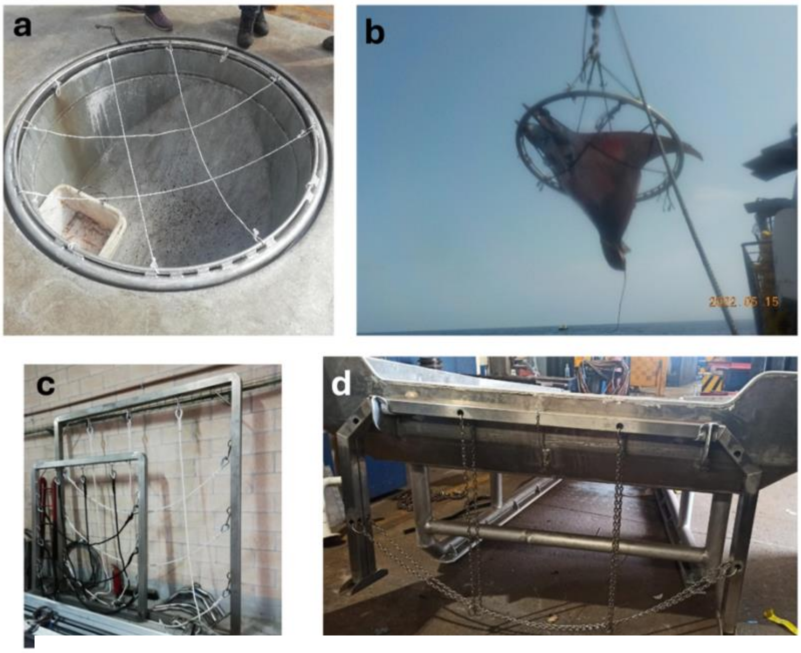

Figure 6. Variations on the manta sorting grid include (a)-(b) circular frames, (c) different sized frames, and (d) foldable frames.

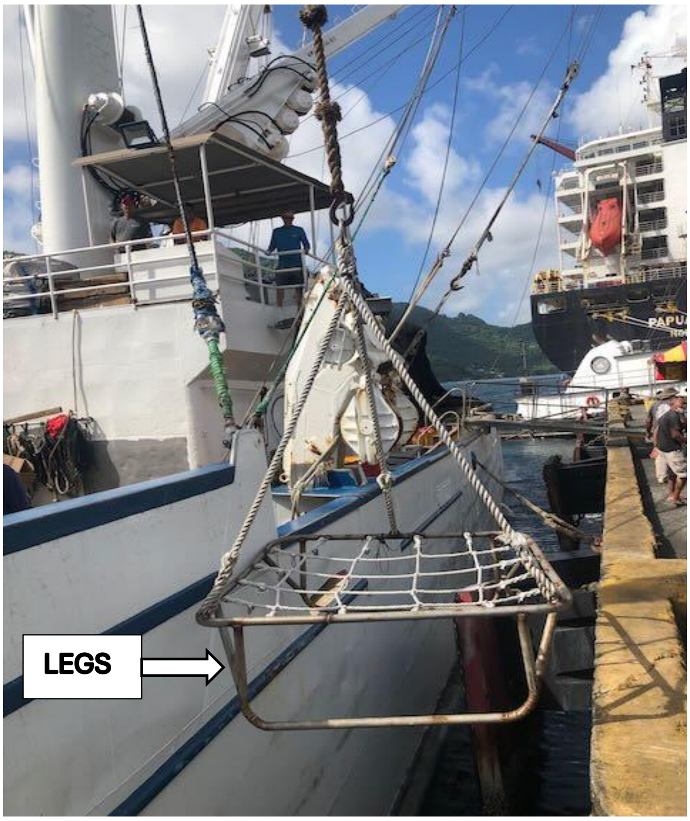

Figure 7. Supporting legs used to prevent the manta sorting grid from resting directly on the hopper, creating a gap that allows tuna to pass through. Photos: ISSF/Western Pacific fisheries, Inc.

Release method variation

Some fishers have devised ideas to release mobulids over the water without the need to cut one of the four lifting ropes. Instead, they use a system with two metallic rings, where one ring is inserted in the crane's hook and another one through which a rope passes. As the rope is pulled up, the rings get closer until they tilt the crane's hook and the metallic loop inside it falls out, thus releasing the animal **(Figure 8).**

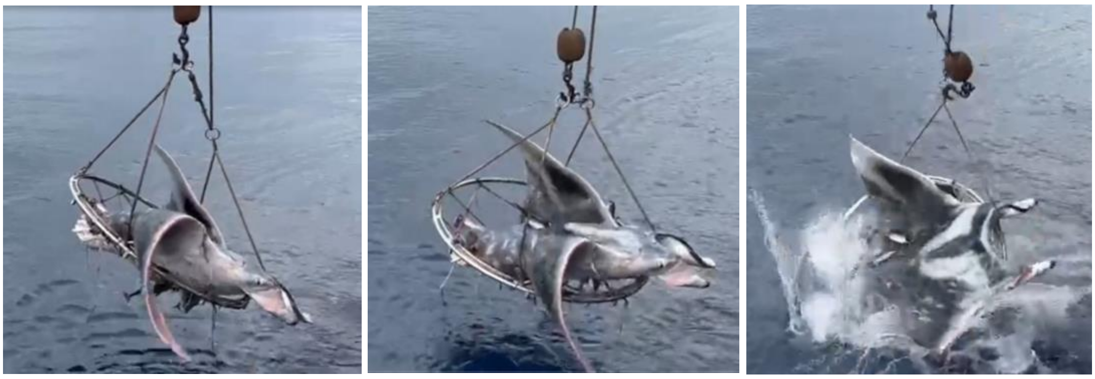

Figure 8. Mobulid release over the water using a grid lifting system with ropes and metallic rings. Photos: AZTI/Echebastar

By incorporating manta sorting grids into standard operating procedures, vessels can meet best-practice handling guidelines, comply with RFMO measures, reduce bycatch mortality, and avoid harmful contact with sensitive anatomical features while maintaining operational efficiency.

References

1. Croll, D. A. et al. Vulnerabilities and fisheries impacts: the uncertain future of manta and devil rays. Aquat. Conserv. Mar. Freshw. Ecosyst. 26, 562–575 (2016).
2. Stewart, J. D. et al. Get them off the deck: Straightforward interventions increase post-release survival rates of manta and devil rays in tuna purse seine fisheries. Biol. Conserv. 299, 110794 (2024).
3. Murua, J. et al. Selective sorting grids for improved best handling and release practices of large mobulid rays in tropical tuna purse seiners. (2024).
4. Murua, J. et al. Incorporating bycatch release devices in guidelines for best bycatch handling and release practices in tropical tuna purse seiners. (2024).
5. Cronin, M.R. et al. Evidence for a fisher-designed solution to manta and devil ray bycatch in tuna fisheries. Conserv. Biol. *In press.*

Appendix

Figure S1. Diagram showing a manta sorting grid release operation (AZTI ©).

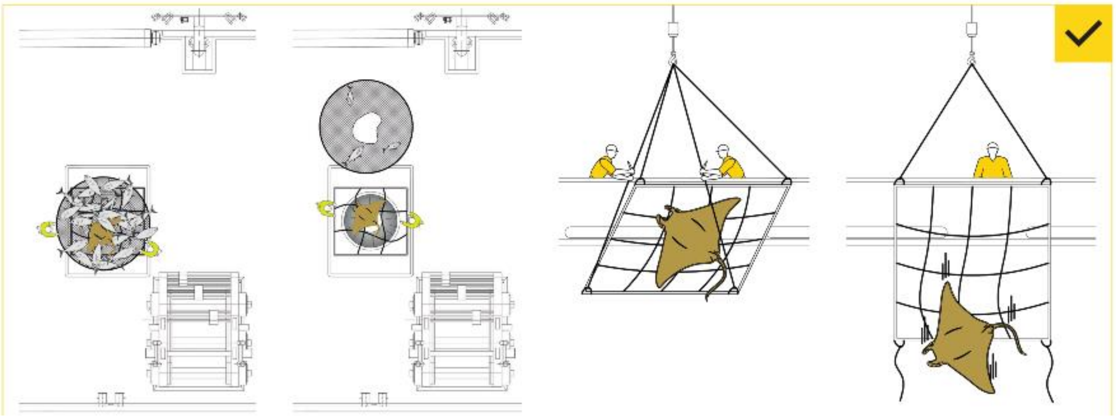

Supplement: Supplementary file 1 — Supporting Information [file COBI-40-e70150-s001.pdf]
