# Supplementary material for: Evidence for a fisher‐designed solution to manta and devil ray bycatch in tuna fisheries
Source: Conserv Biol. 2025 Oct 22;40(1):e70150. doi: 10.1111/cobi.70150 (PMC12856801; doi:10.1111/cobi.70150)
Supplement: Supplementary file 2 — Supporting Information [file COBI-40-e70150-s002.docx]

**Appendix S1**

**Evidence for a fisher-designed solution to manta and devil ray bycatch in tuna fisheries**

This document was prepared by Jefferson Murua (AZTI), for the mobulid sorting grid trial project led by the International Seafood Sustainability Foundation, UC Santa Cruz and NOAA’s Pacific Island Regional Office.

**INFORMATION ON MANTA RAY SORTING GRIDS**

In the past moving large adult mantas arriving on the vessel’s deck back into the water, which is not an easy task, was conducted with poor practices such as lifting them with ropes or hooks by gill slits. Now these practices are prohibited by measures adopted by WCPFC (CMM-19-05) and IATTC (C-15-04). It is important to remember that most species of mobulids are in a vulnerable or endangered status, and even if few interactions with purse seiners take place, they still have an effect because of their declining small populations. Also, the more threatened the status of a species, especially if it is a charismatic one like whales or manta rays, the greater the anti-fishing campaigns by environmental NGOs to influence consumer choice.

Several alternatives to release manta rays in a better way have developed in the last years. For example, the use of cargo nets to lift them has become common practice (Figure 1).


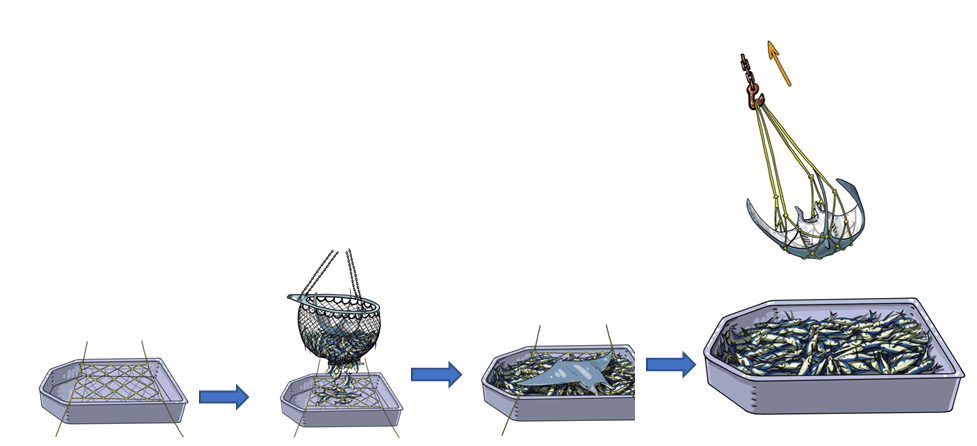


**Figure 1** – Manta release method with cargo net in the hopper (Poisson et al., 2012)

This method is considered simple and much better than old practices, however it is still improvable. For example, the use of cargo nets in most cases still requires that the animal is extracted by hand from the brail or the hopper. This is a risk to crew having to lift such a heavy animal and for the manta itself because it is usually grasped by gills, horns, or wherever they can be held. It also may take a few minutes to extract a manta before starting to lift it, and data shows that every extra minute out of the water has a marked impact on the survival of these aquatic animals. Also, some cargo nets when lifted can result in an excessive folding of the animals’ wings which may cause damage.


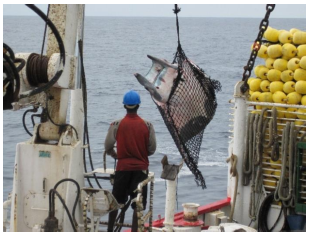


Figure 2. Mobulid lifted in a small cargo net

Recently, based on ideas proposed by experienced skippers, scientists from AZTI have developed sorting grids that try to prevent these release deficiencies. These grids consist simply of a rigid frame (can be square, round, etc.), with a series of crossed ropes or chains that act as filters to let the fish go through while the manta stays on top. It is important to take correct measurements of the unloading hatch or hopper tray, depending on where the brail is emptied, to construct a sorting grid with the correct size.

For a square sorting grid only 4 stainless steel tubes are required. These should be sufficiently robust to withstand the weight of one or more manta rays. A typical frame would be 200 cm length x 6 cm wide x 6 cm height (78 inches x 2.5 inches x 2.5 inches) and if the stainless-steel tube is hollow, it should have a minimum wall of 4 mm (0.16 inches). The length of the frame can vary like in the photo below (Figure 3) depending on the vessel characteristics. If the sorting grid is used on the unloading hatch, it should be larger than the hole and if it is used on the hopper, it should be narrower to fit inside.

On the inner part of each side of the frame there should be 3-4 welded half rings with approx. 25 cm (10 inches) separation to provide attachment points for the ropes that will conform the grid cells. In addition, in each of the inner corners of the frame having a welded bar (see Figure 5) will help as an attachment point for the 4 ropes that will lift it with the crane.


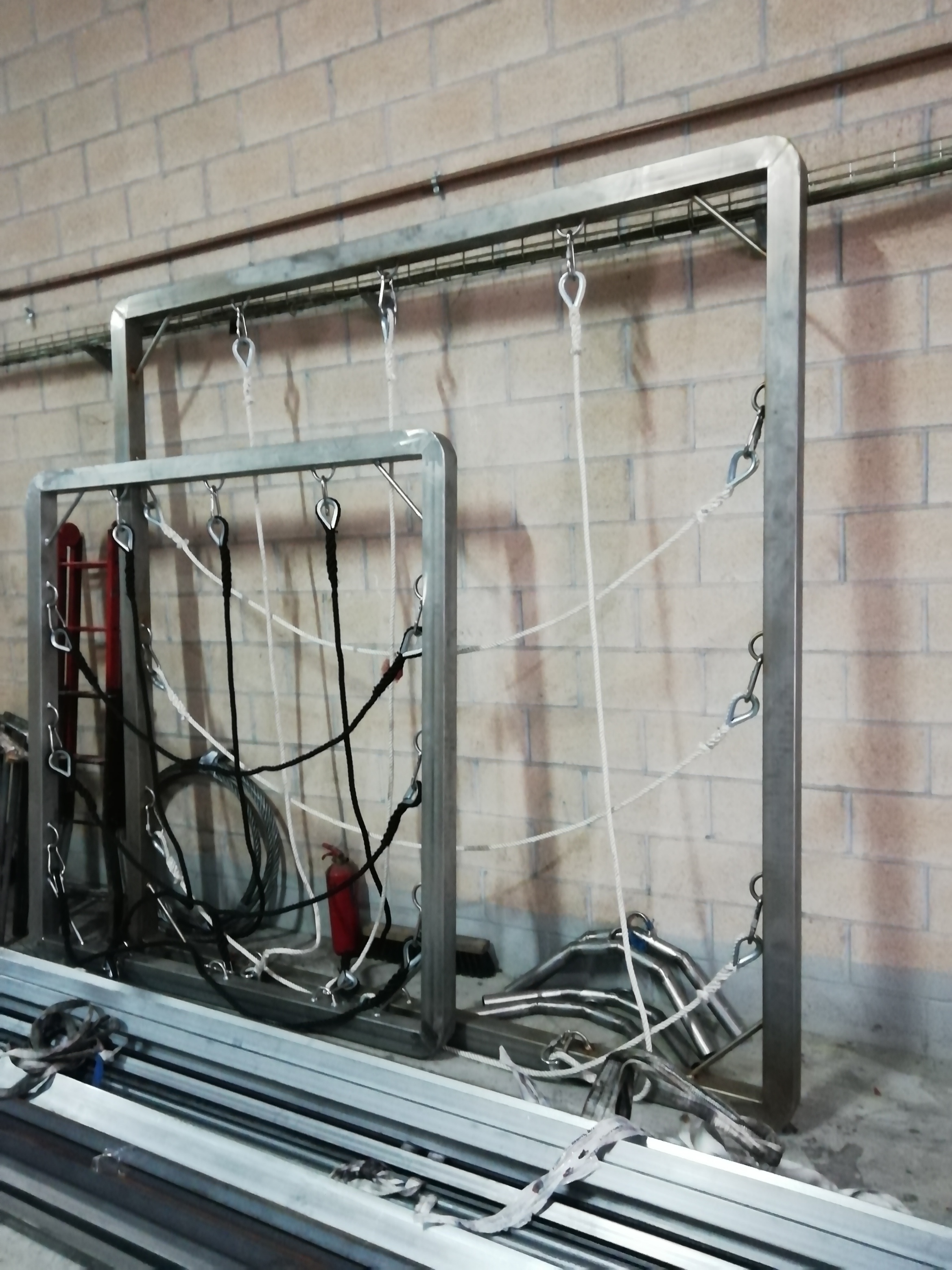


Figure 3. Sorting grids of different sizes


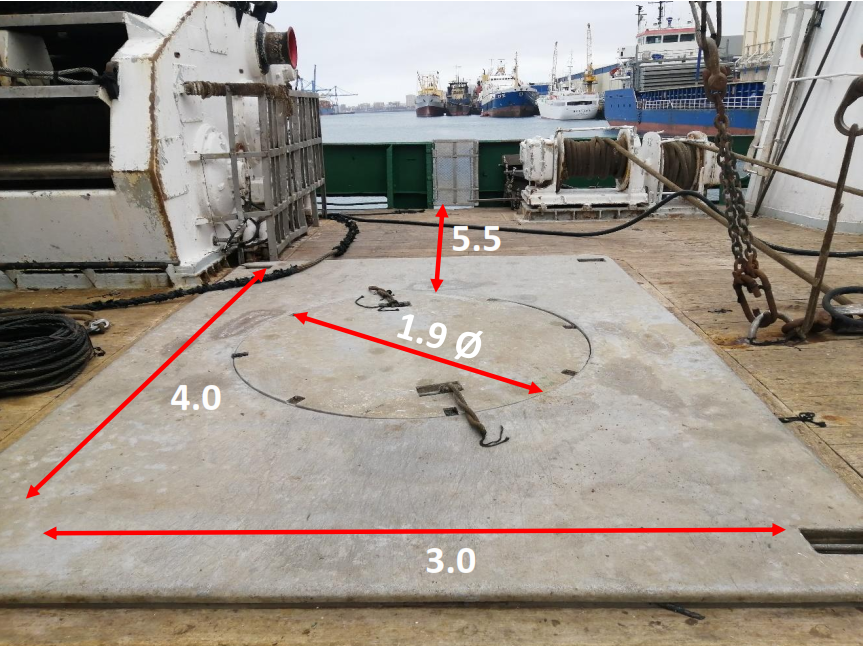


Figure 4. Unloading hatch measurements (in meters) and distance to starboard door. The frame of the grid should be larger than the diameter of the hole.

These sorting grids are either put directly on top of the unloading hatch or for vessels with hoppers inside the hopper’s tray. Experiments with the grids have shown that manta rays can be released in 1-2 minutes, without need for the fishers to handle it manually. Meanwhile, the brailer is not held back and can continue to work normally.


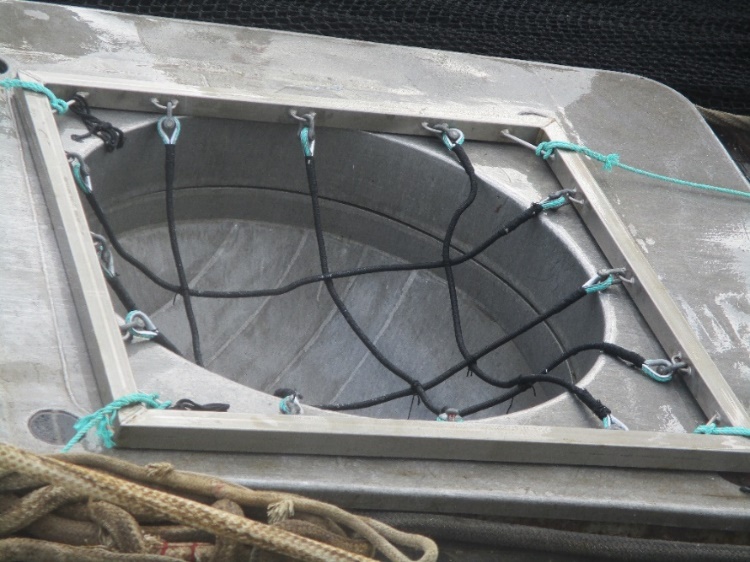


Figure 5. Manta ray sorting grid on unloading hatch with a 3 x 3 rope configuration

1.
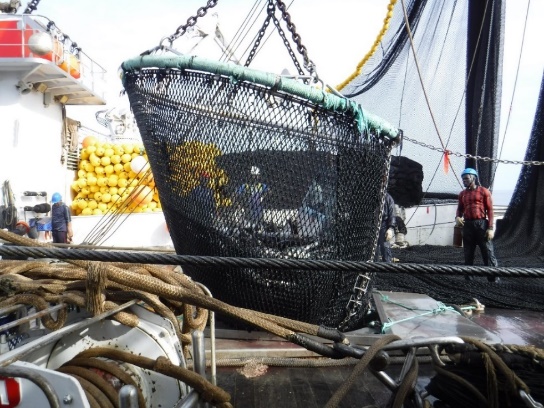
 (b)
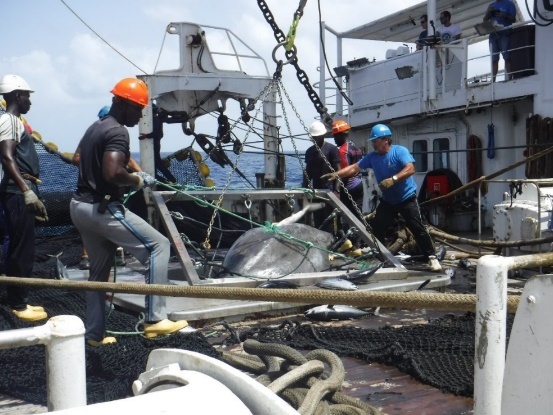


(c)
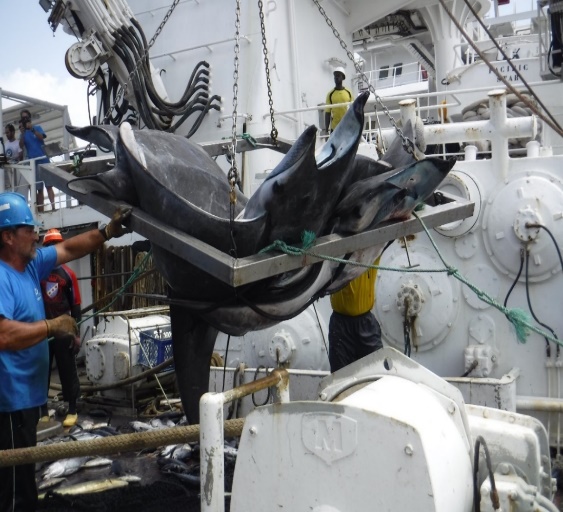
 (d)
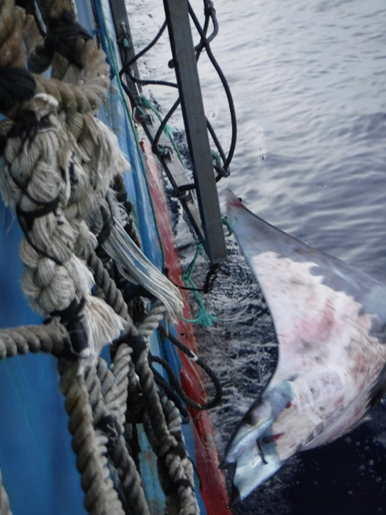


Figure 6. Manta ray sorting grid release steps: (1) empty brail contents into unloading hatch, (2) connect grid frame with chains to deck crane, (3) move sorting grid towards starboard railing, and (4) release rays into the water.

Alternative sorting grids have been prepared to fit in the unloading hatch rim (i.e., round sorting grids) as well.


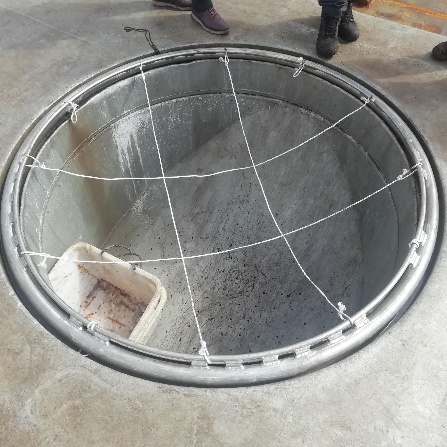

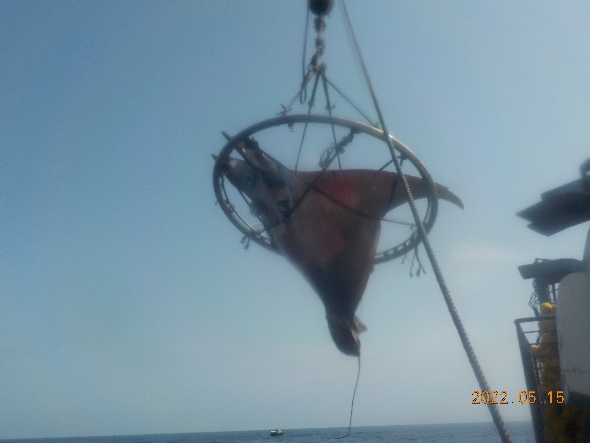


Figure 7. Round mobulid sorting grid to fit in the unloading hatch perimeter


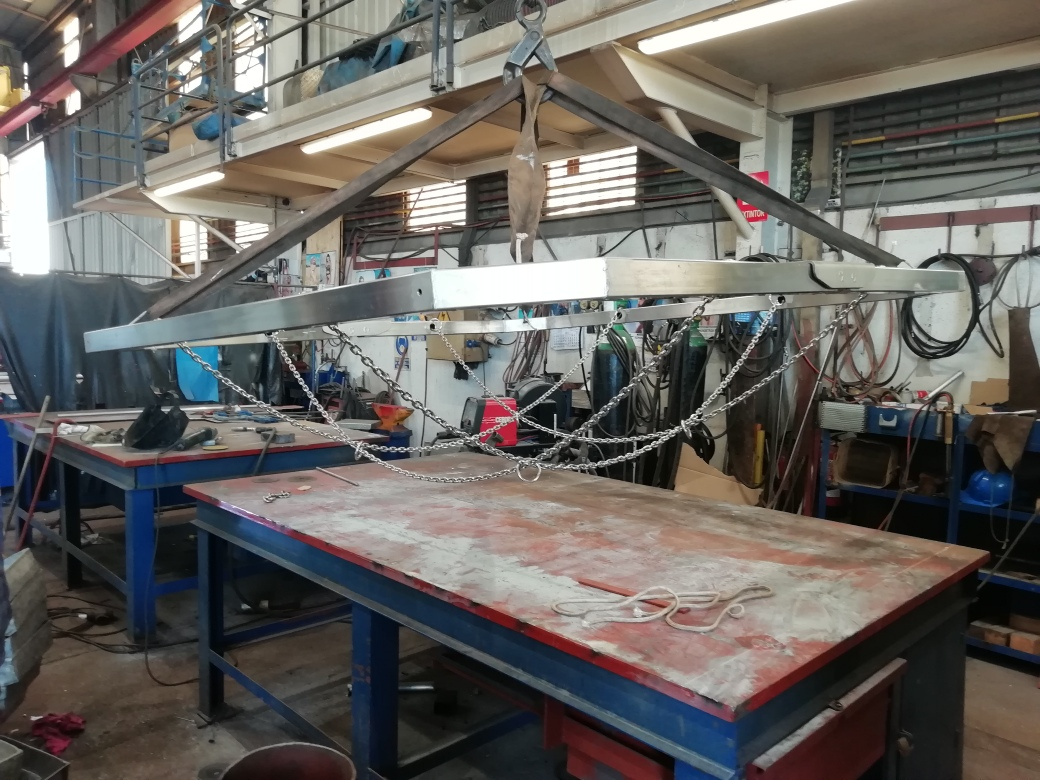

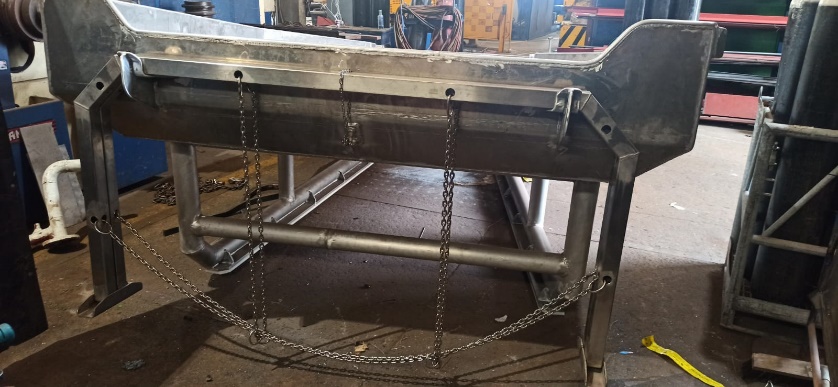


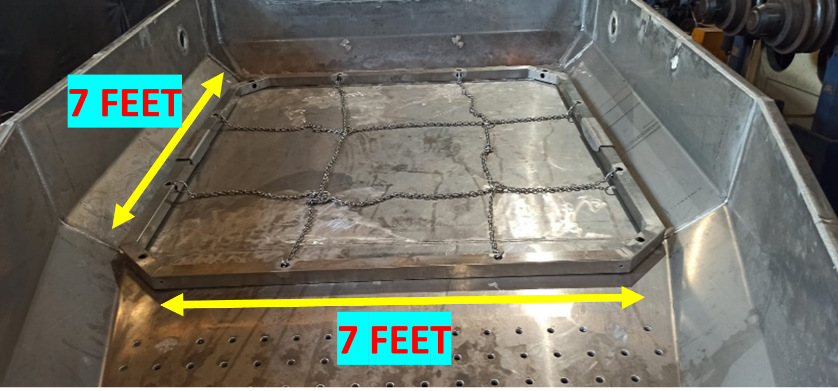


Figure 8. Foldable sorting grid storable behind the hopper

As it can be seen different designs are possible and can be suited to the requirements of each vessel’s circumstances. It is important to know how each vessel works, which deck distribution they have to better design the sorting grid design and protocol, so it interferes the least possible with the brailing operation on deck

**Information requested from your vessel:** Requirements are different depending on the equipment on deck with and without hoppers. Photos showing the deck space and hopper designs are welcome.

1. **Vessels WITHOUT hopper**

- Would it be possible to conduct the protocol shown in Figure 6 of positioning a sorting grid on the unloading hatch and lift with the cargo crane all the way to the starboard?
- Are there any obstacles on the starboard (e.g., davits, workboats, etc.) preventing the cargo crane from taking the sorting grid all the way to the water’s edge?
- Is the unloading hatch opening round or square? What are the exact measurements (see figure 4)?
- Is the brailer always unloaded on the same unloading hatch or there are several openings distributed across the deck?
- Is there any other information you think is relevant to conduct this kind of release operation?

1. **Vessels WITH hopper**

- Would it be possible to conduct the protocol shown in Figure 1 moving the manta directly from the hopper to the starboard, but instead of using a cargo net using a sorting grid?
- If not, are there other ways in which the manta in the sorting grid could be transported directly (without lifting the manta manually out of the hopper) to the starboard or to the portside for release?
- Are there any obstacles on the starboard (e.g., davits, workboats, etc.) preventing the cargo crane from taking the sorting grid all the way to the water’s edge?
- What are the exact dimensions of your hopper in terms of width and length? (to estimate the size of the sorting grid that could fit inside like in figure 8)
- Is there any other information you think is relevant to conduct this kind of release operation?
